# Supplementary material for: Triflamidation of Allyl-Containing Substances:Unusual Dehydrobromination vs. Intramolecular Heterocyclization
Source: Molecules. 2022 Oct 14;27(20):6910. doi: 10.3390/molecules27206910 (PMC9610413; doi:10.3390/molecules27206910)

Supporting Information for

Triflamidation of Allyl-Containing Substances. Unusual Dehydrobromination vs. Intramolecular Heterocyclization

Anton S. Ganin, Mikhail Yu. Moskalik, Ivan A. Garagan, Vera V. Astakhova, Bagrat A. Shainyan

Figure S1. ^1H NMR spectrum of compound **4**

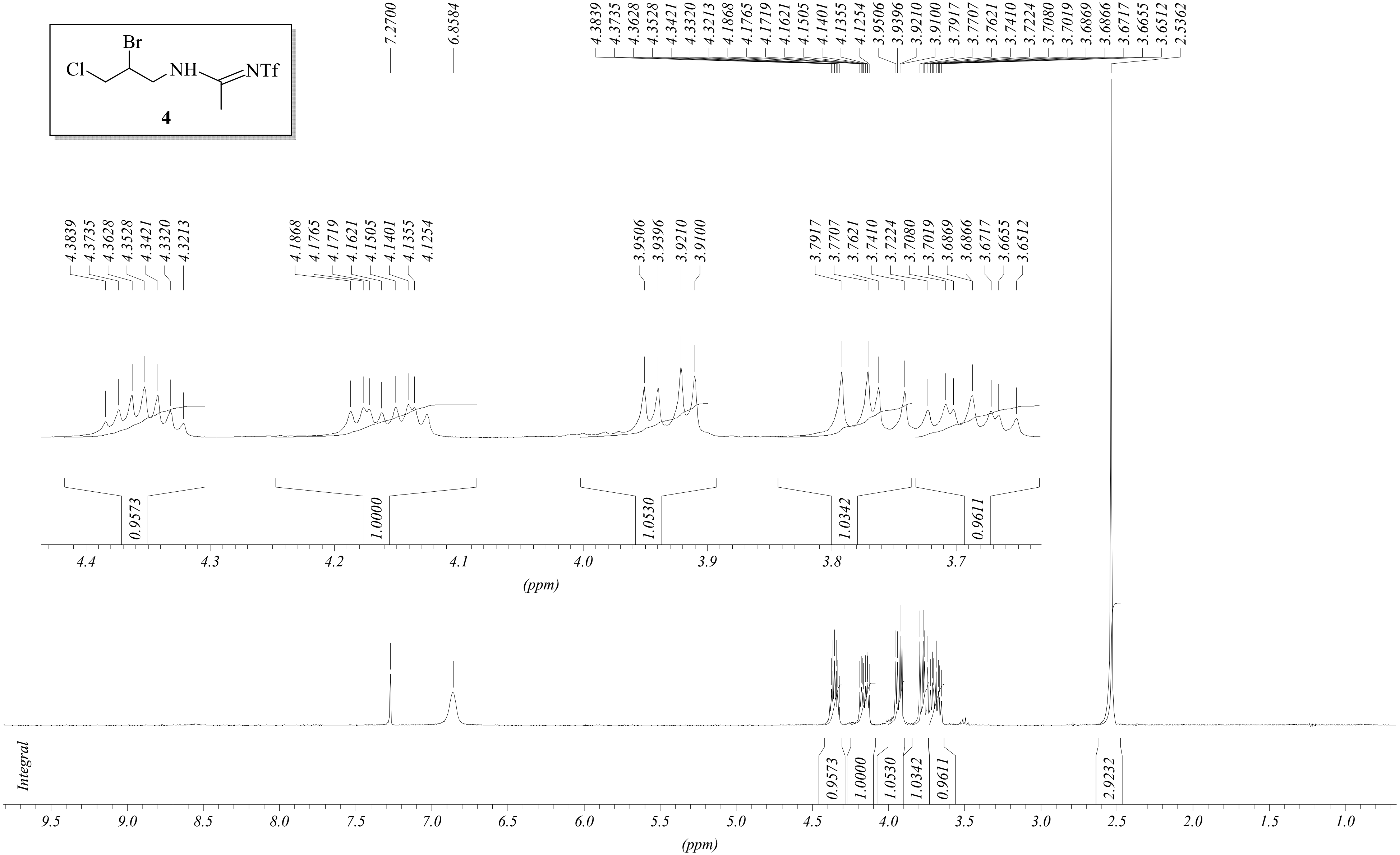

Figure S2. ^{13}C NMR spectrum of compound **4**

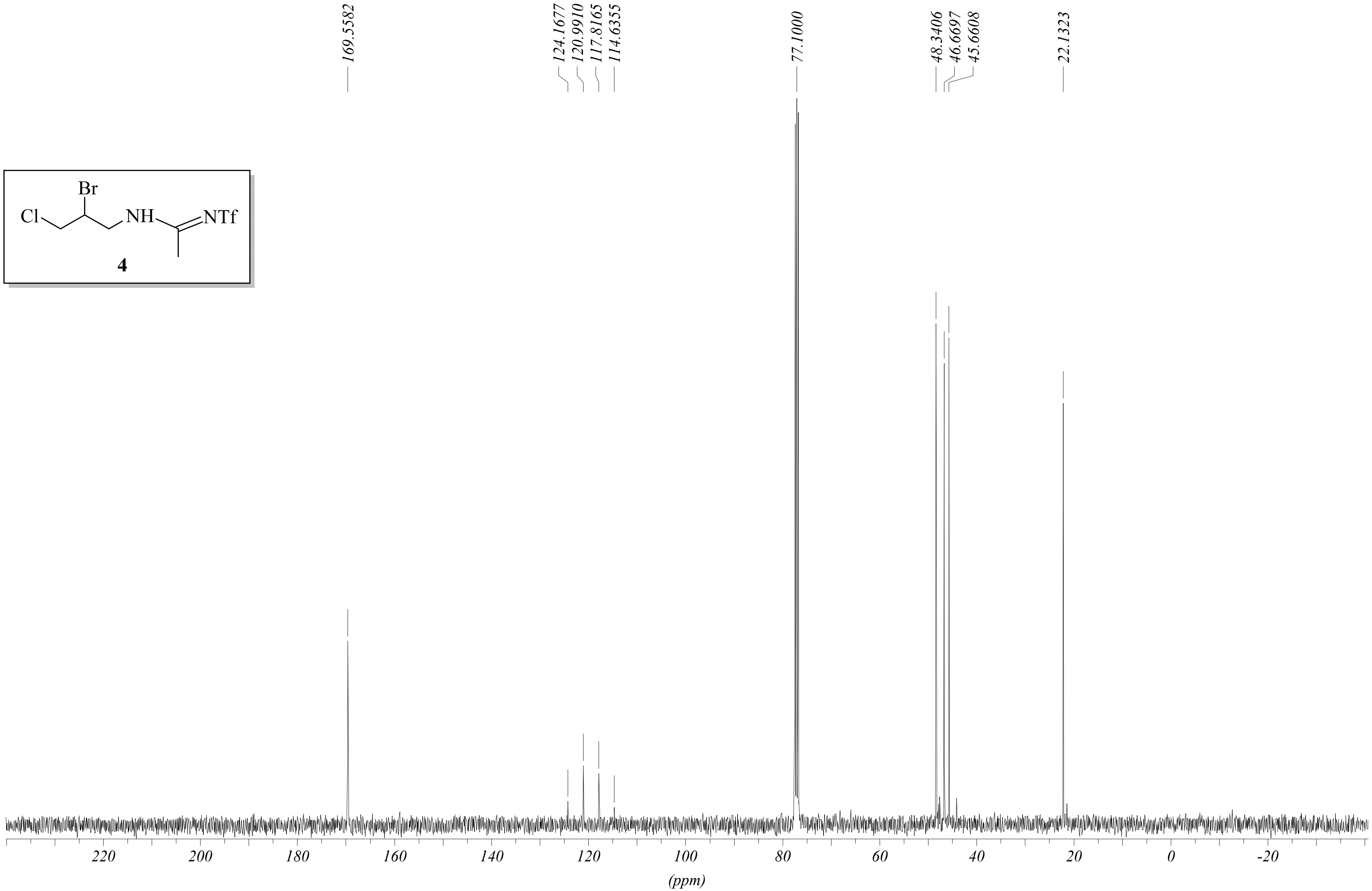

Figure S3. ^{13}C (J-mod) NMR spectrum of compound **4**

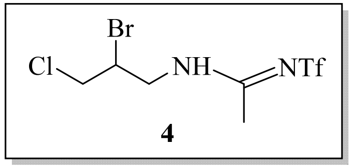

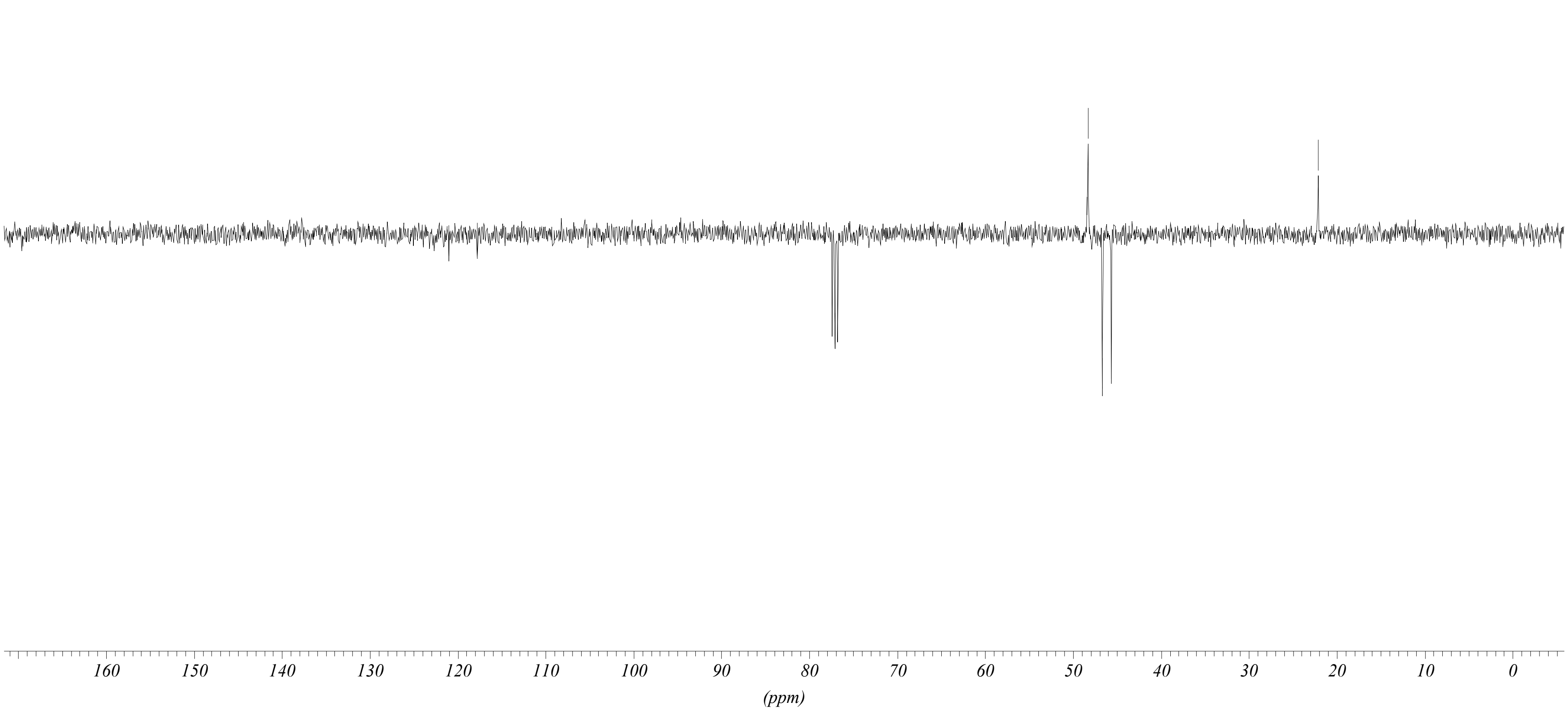

Figure S4. ^{1}H NMR spectrum of compound **5**

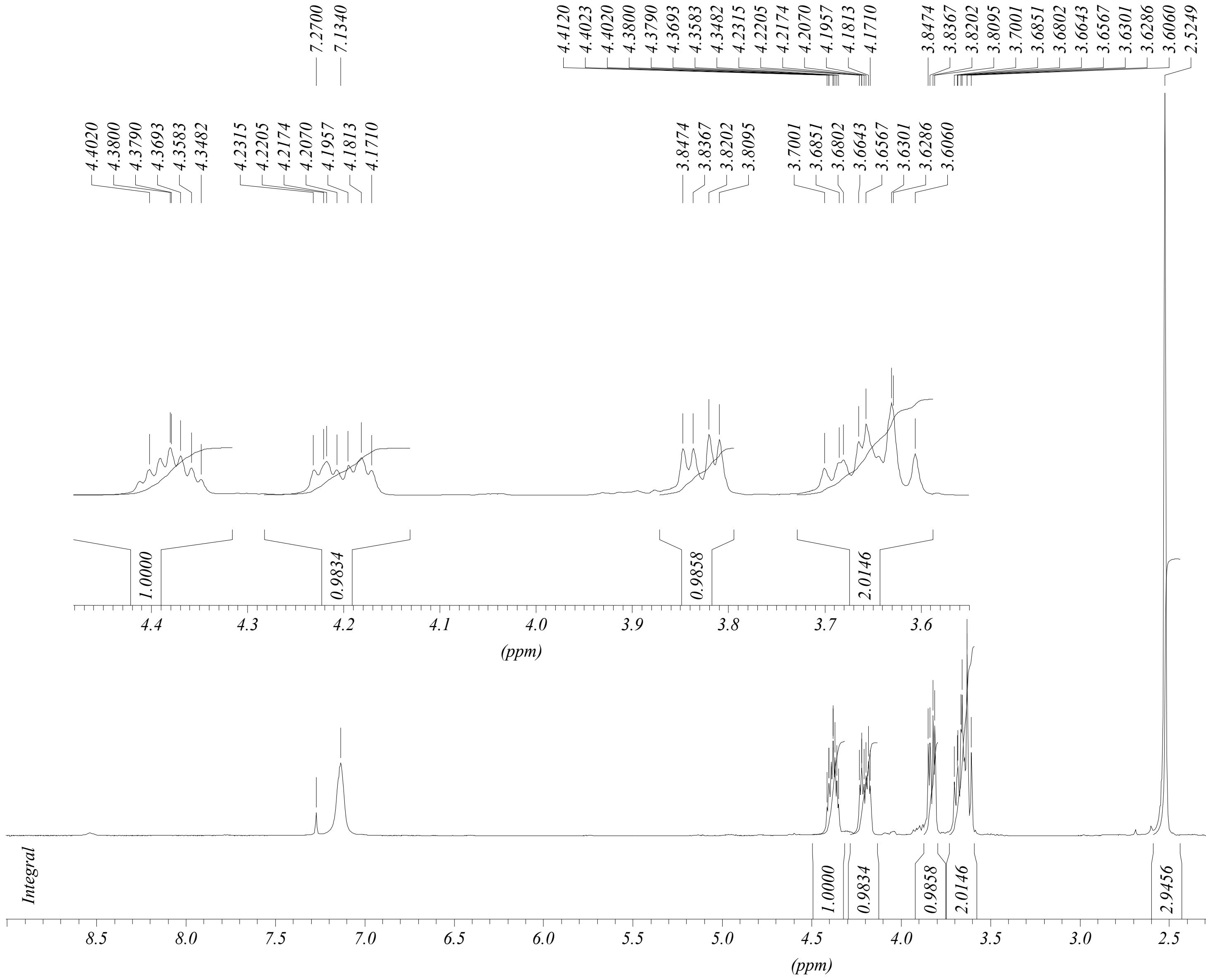

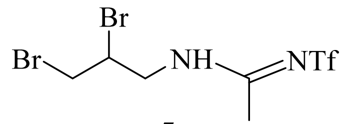

5

Figure S5. ^{13}C NMR spectrum of compound **5**

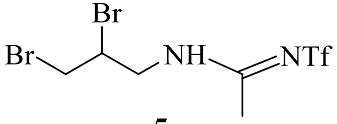

5

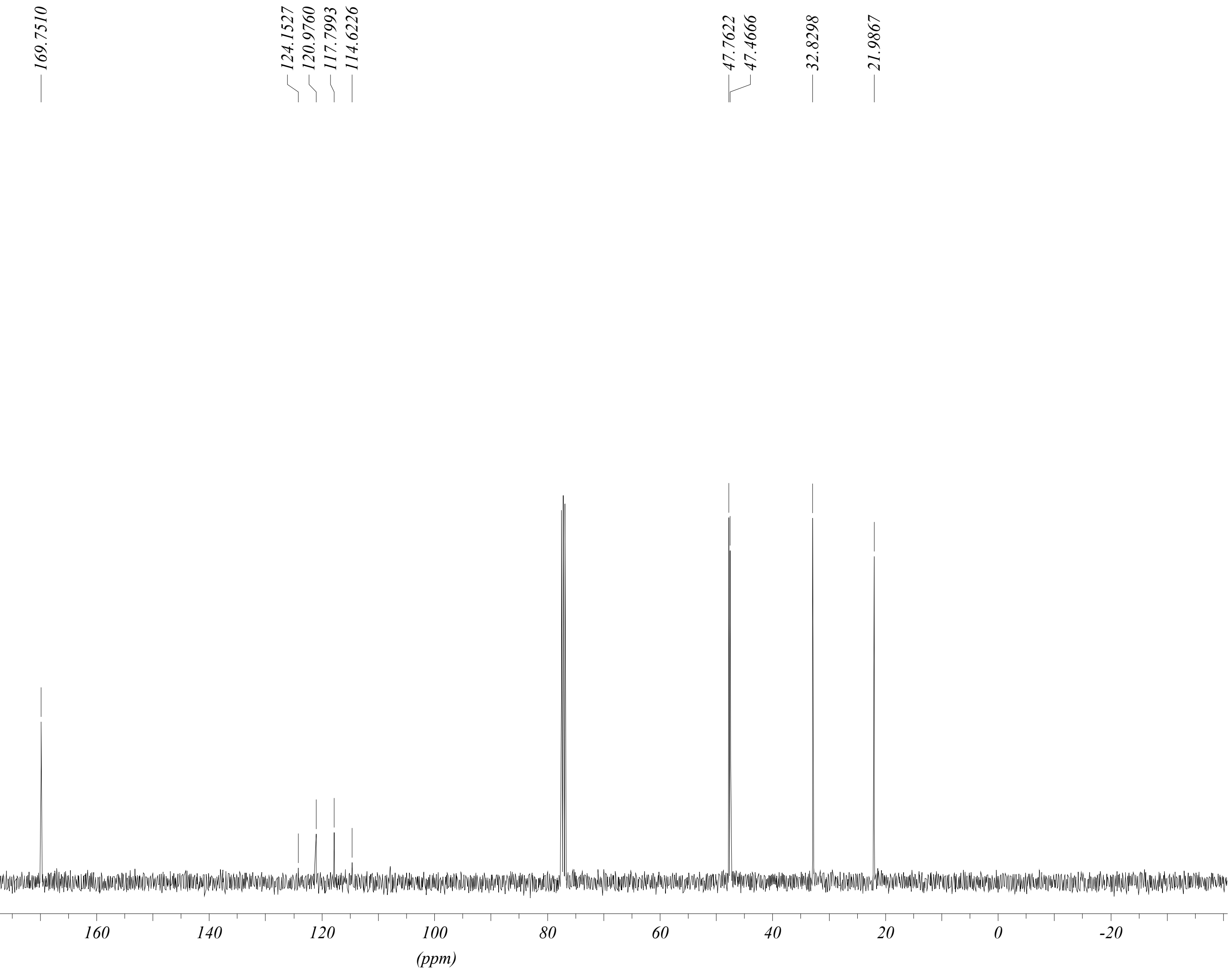

Figure S6. ^{1}H NMR spectrum of compound **8**

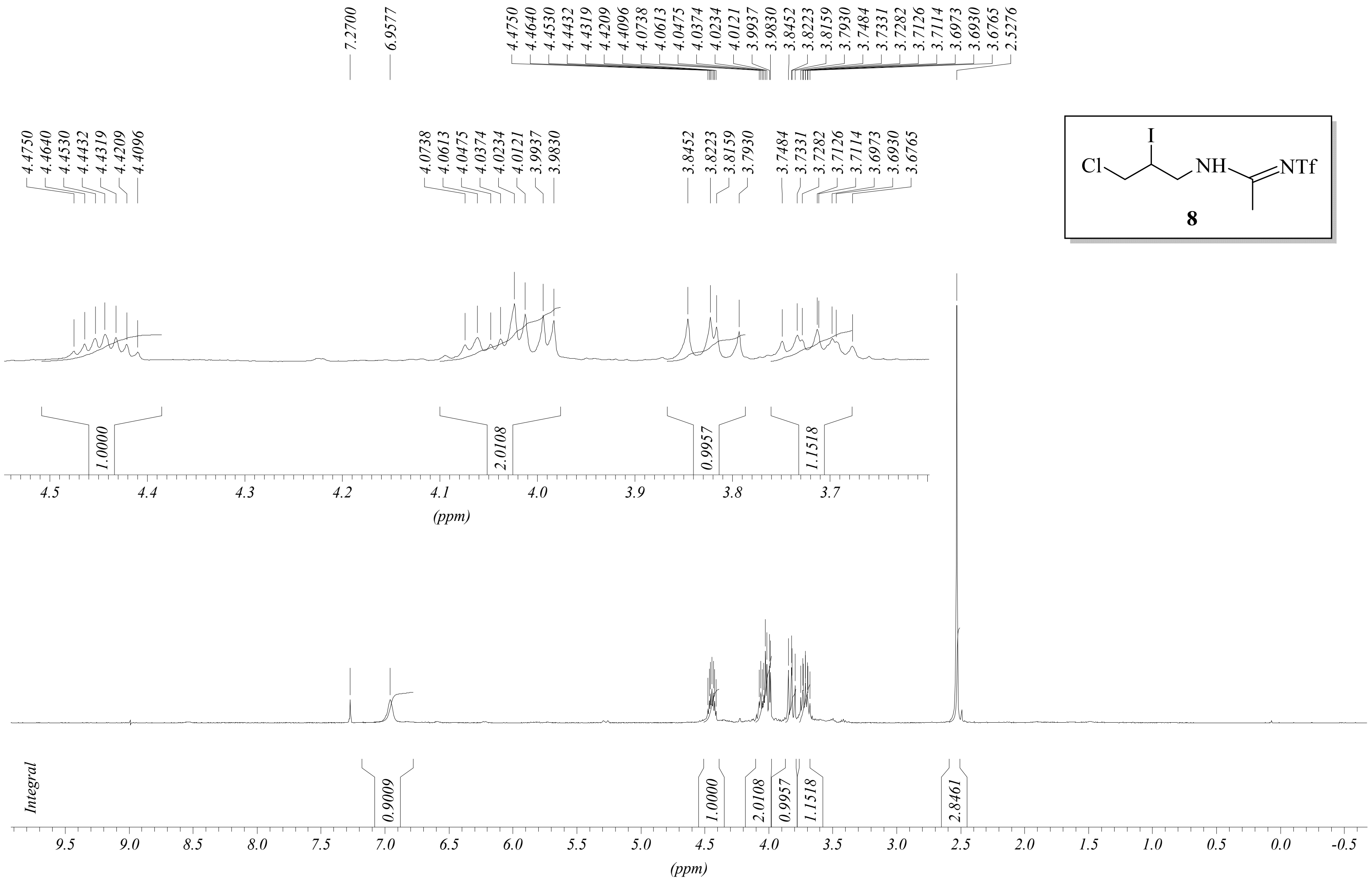

Figure S7. ^{13}C NMR spectrum of compound **8**

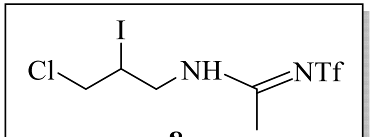

8

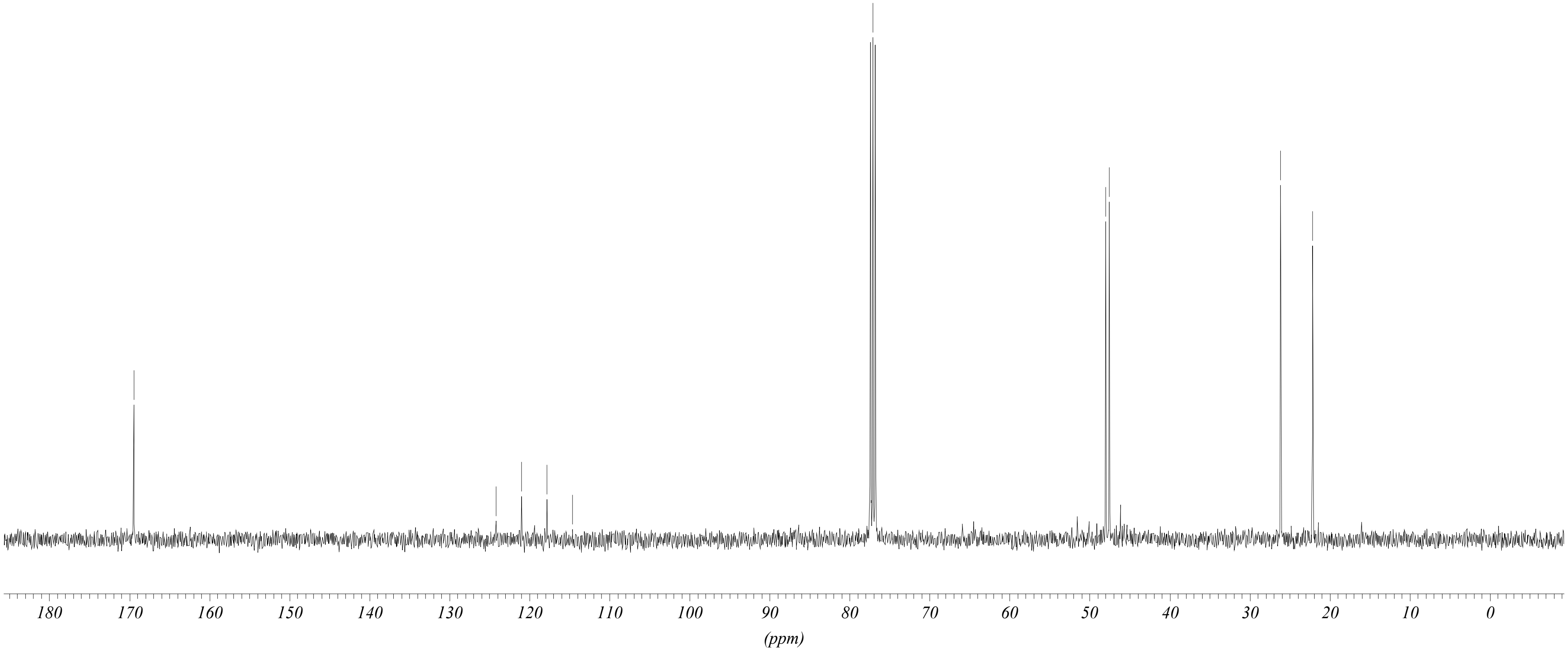

Figure S8. ^{13}C (J-mod) NMR spectrum of compound **8**

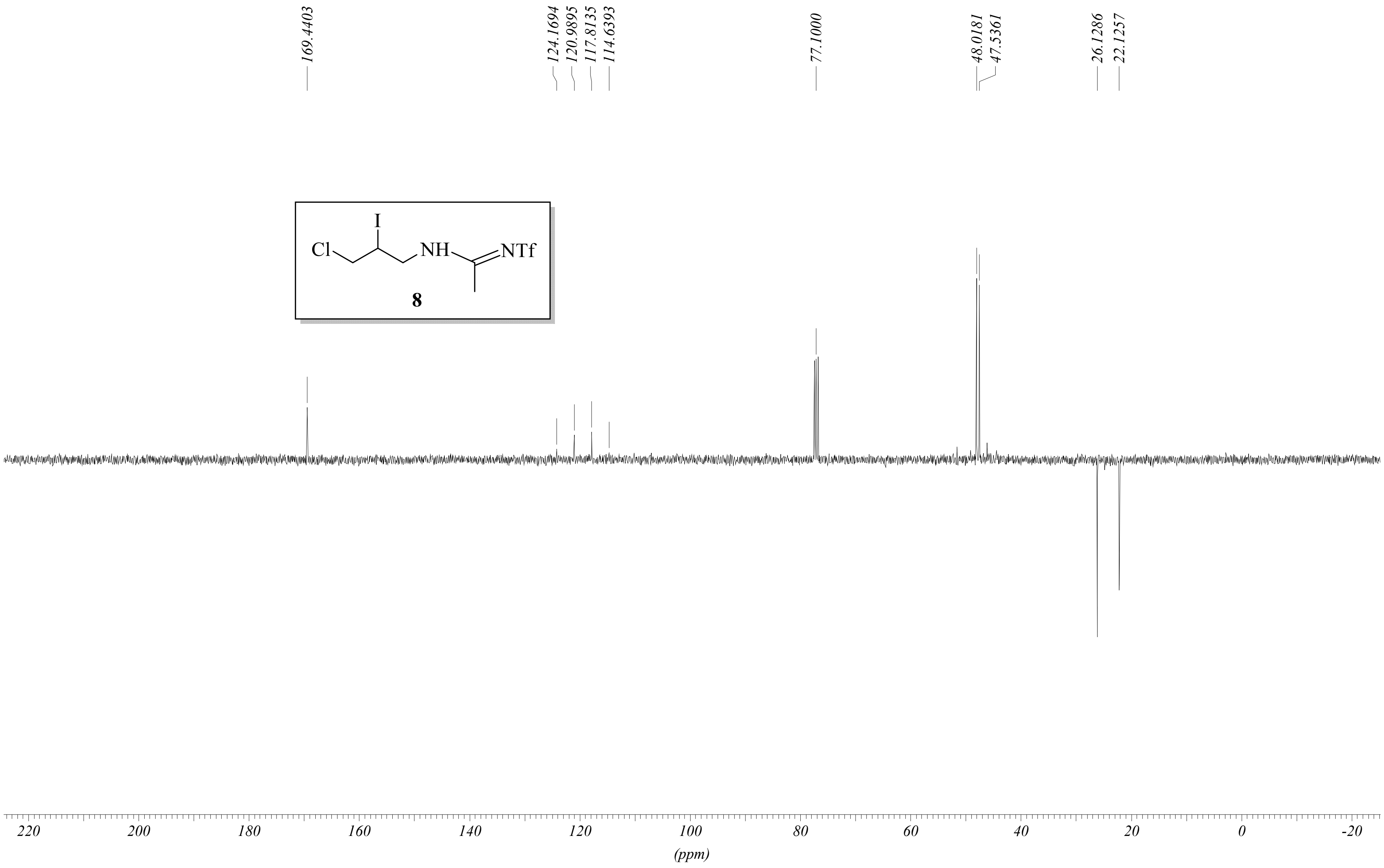

Figure S9. ^{13}C ([13C-1H]) NMR spectrum of compound **8**

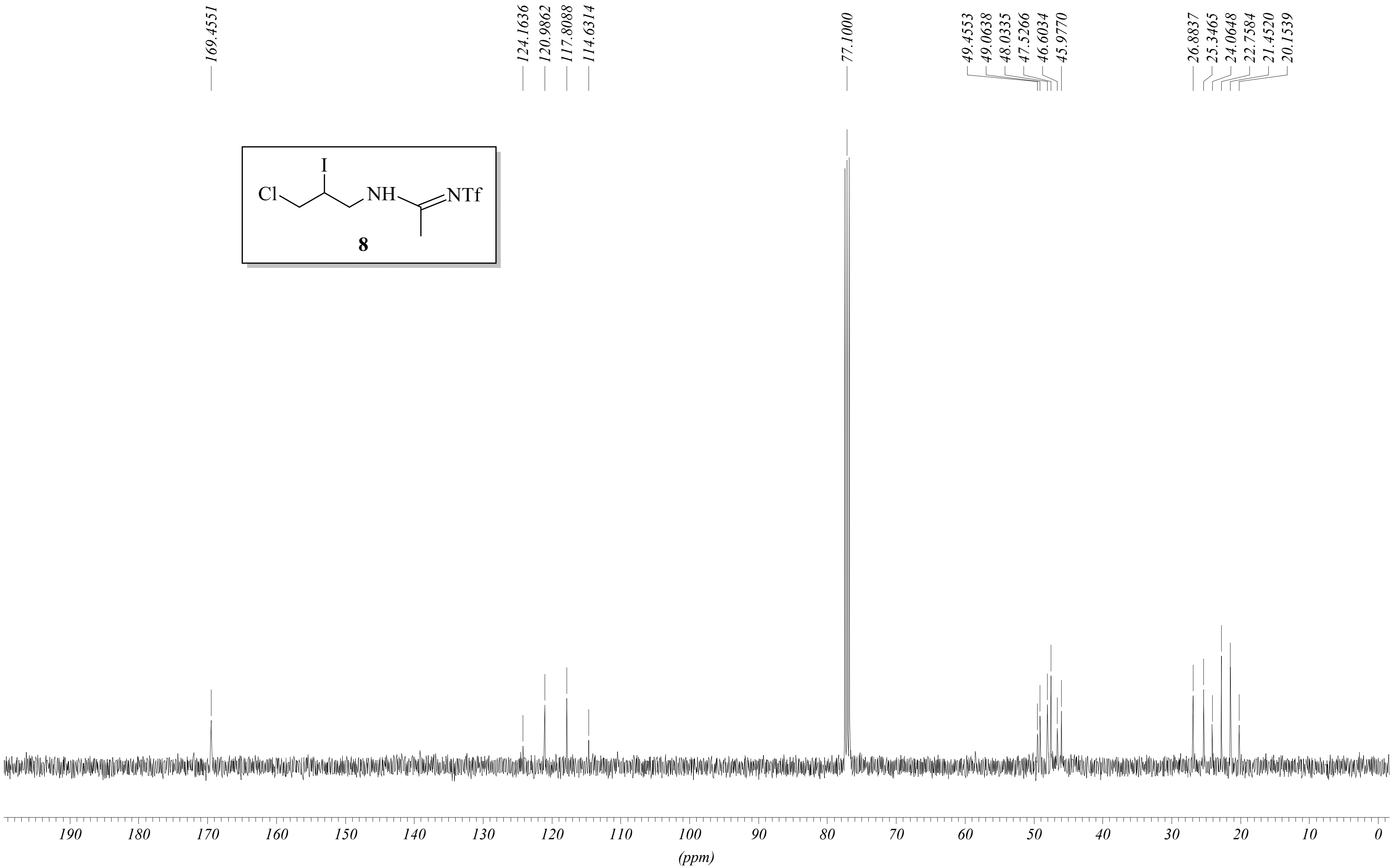

Figure S10. ^{1}H NMR spectrum of compound **9**

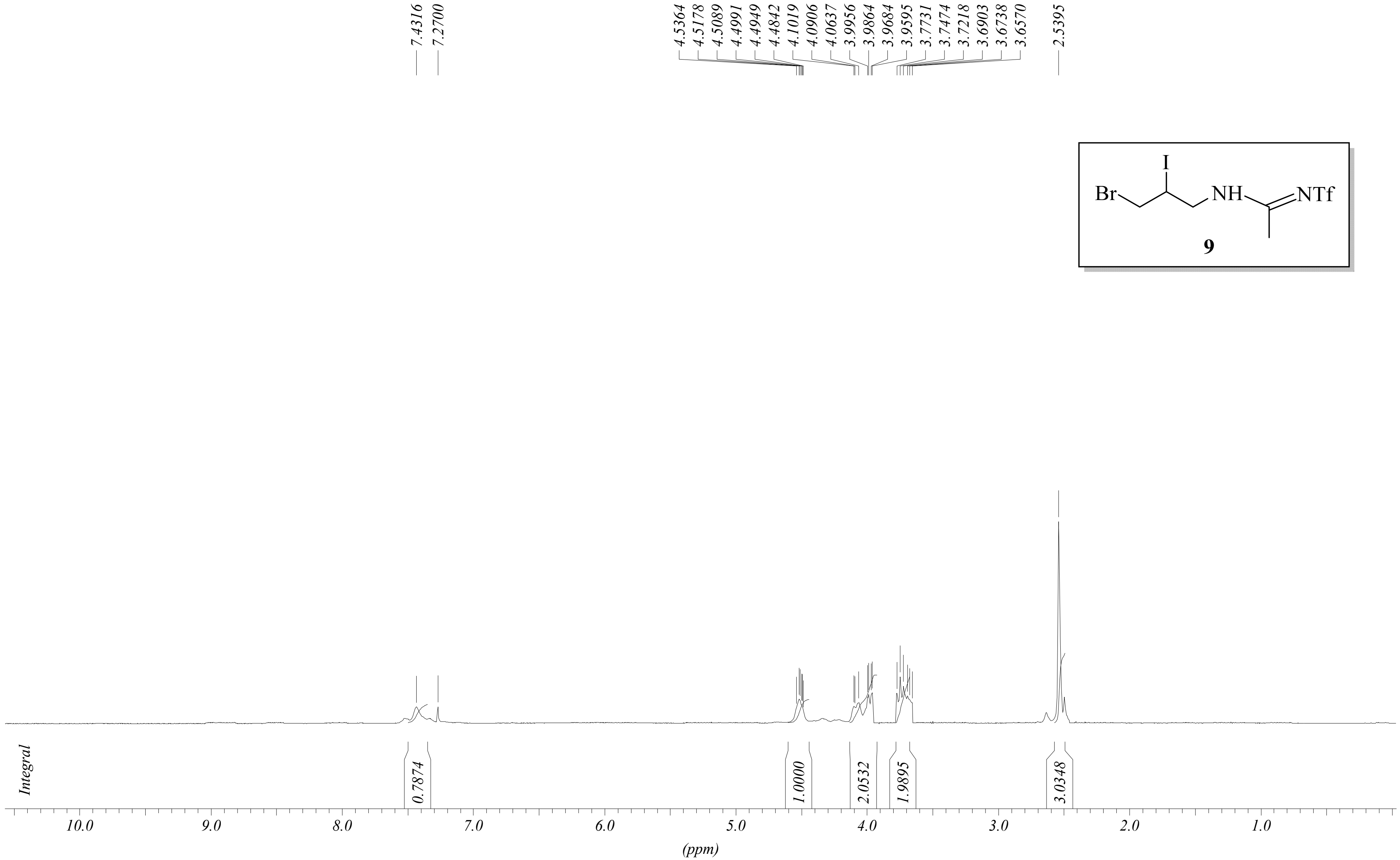

9

Figure S11. ^{13}C NMR spectrum of compound **9**

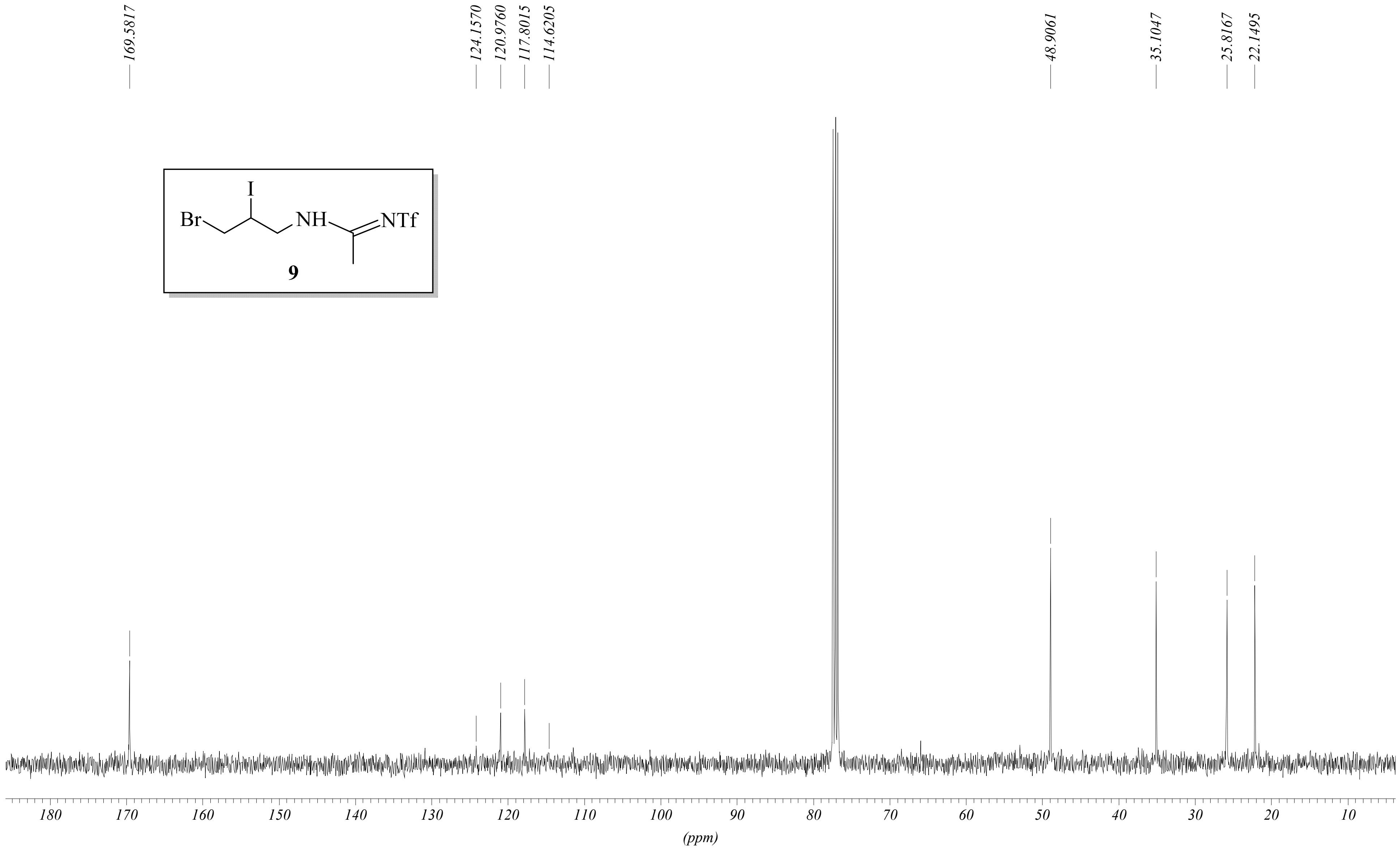

Figure S12. ^{1}H NMR spectrum of compound **14**

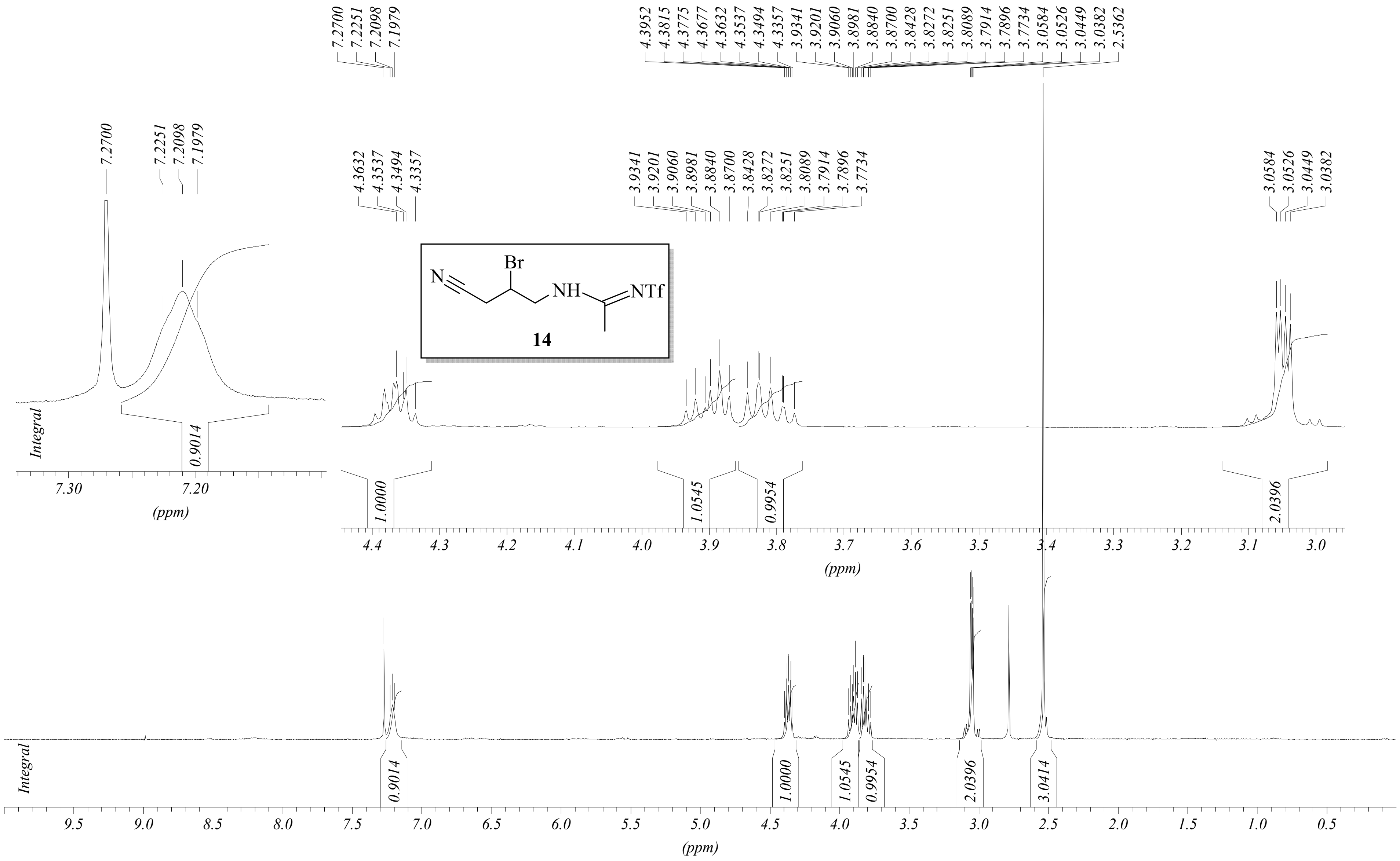

Figure S13. ^{13}C NMR spectrum of compound **14**

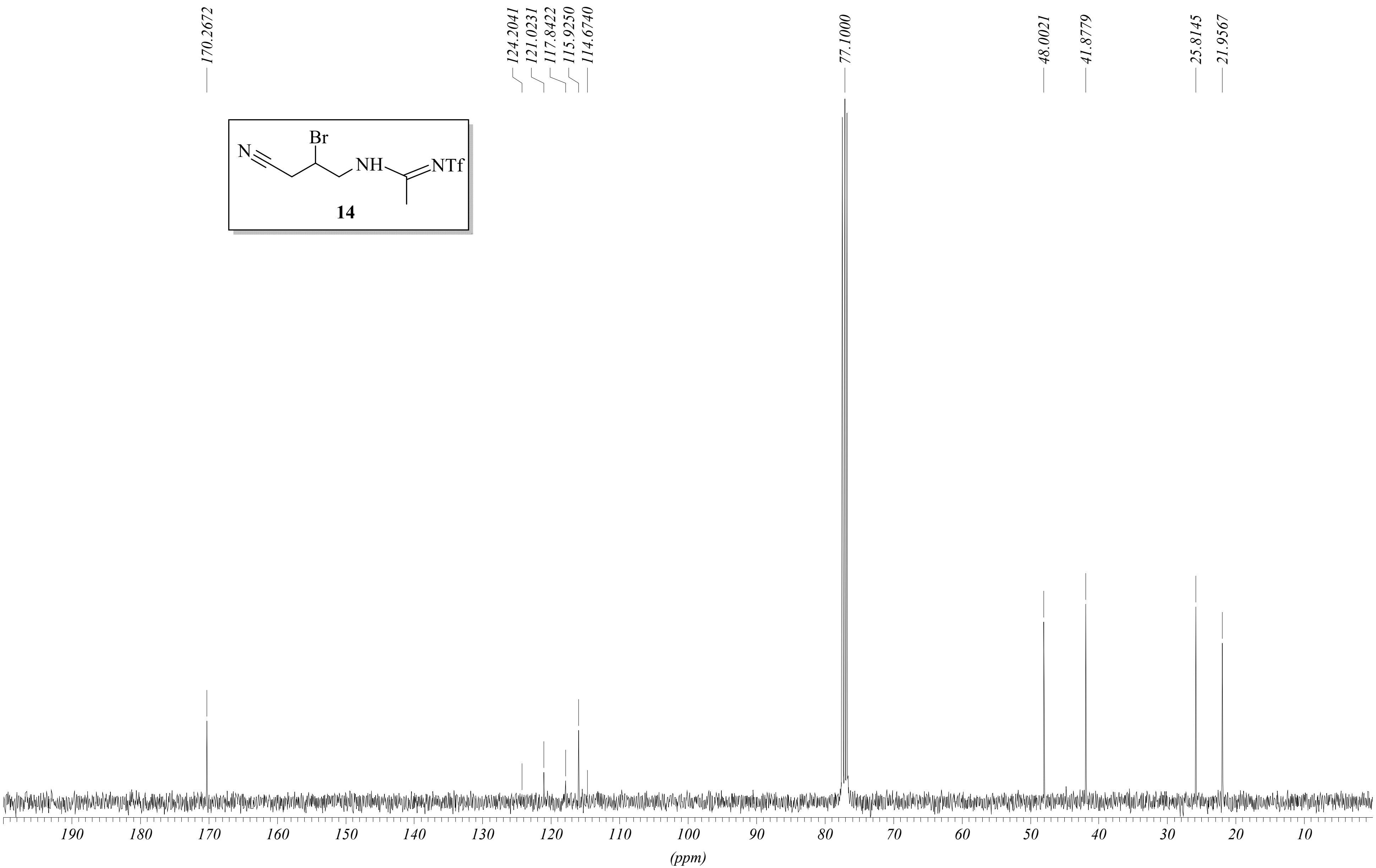

Figure S14. ^{1}H NMR spectrum of compound **15** and **18**

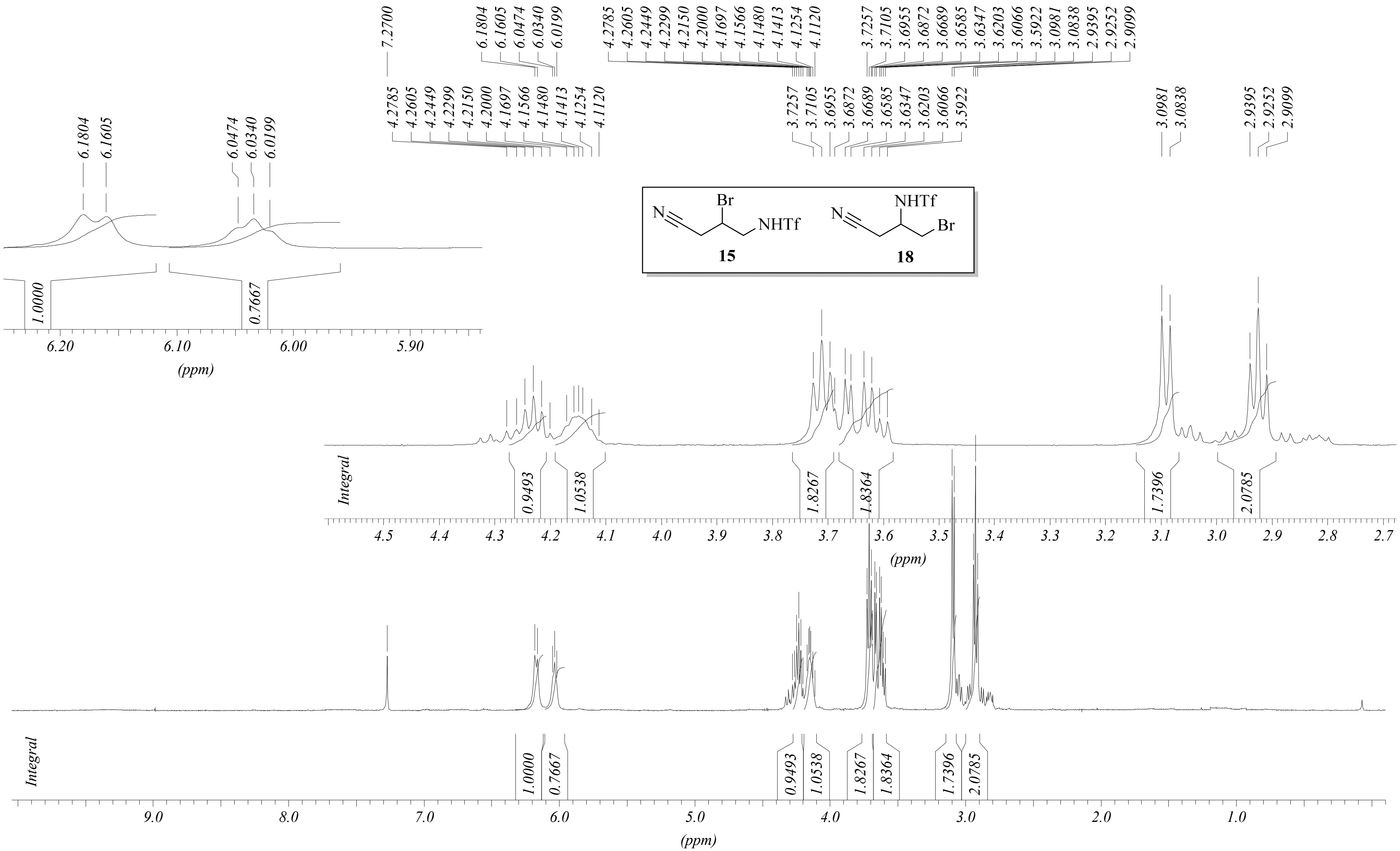

Figure S15. ^{13}C (J-mod) NMR spectrum of compound **15** and **18**

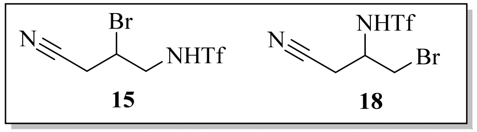

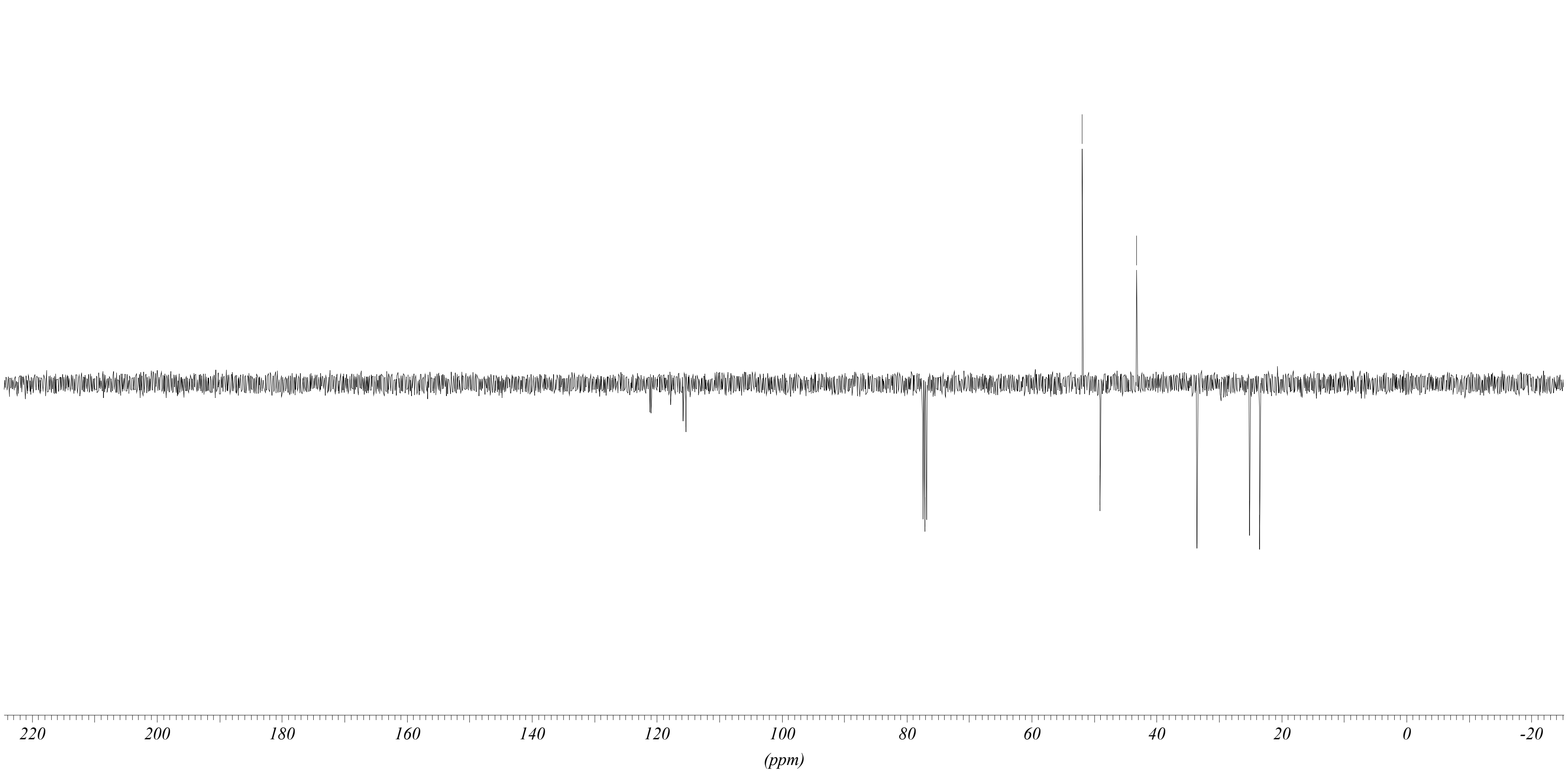

Figure S16. ^{13}C NMR spectrum of compound **15** and **18**

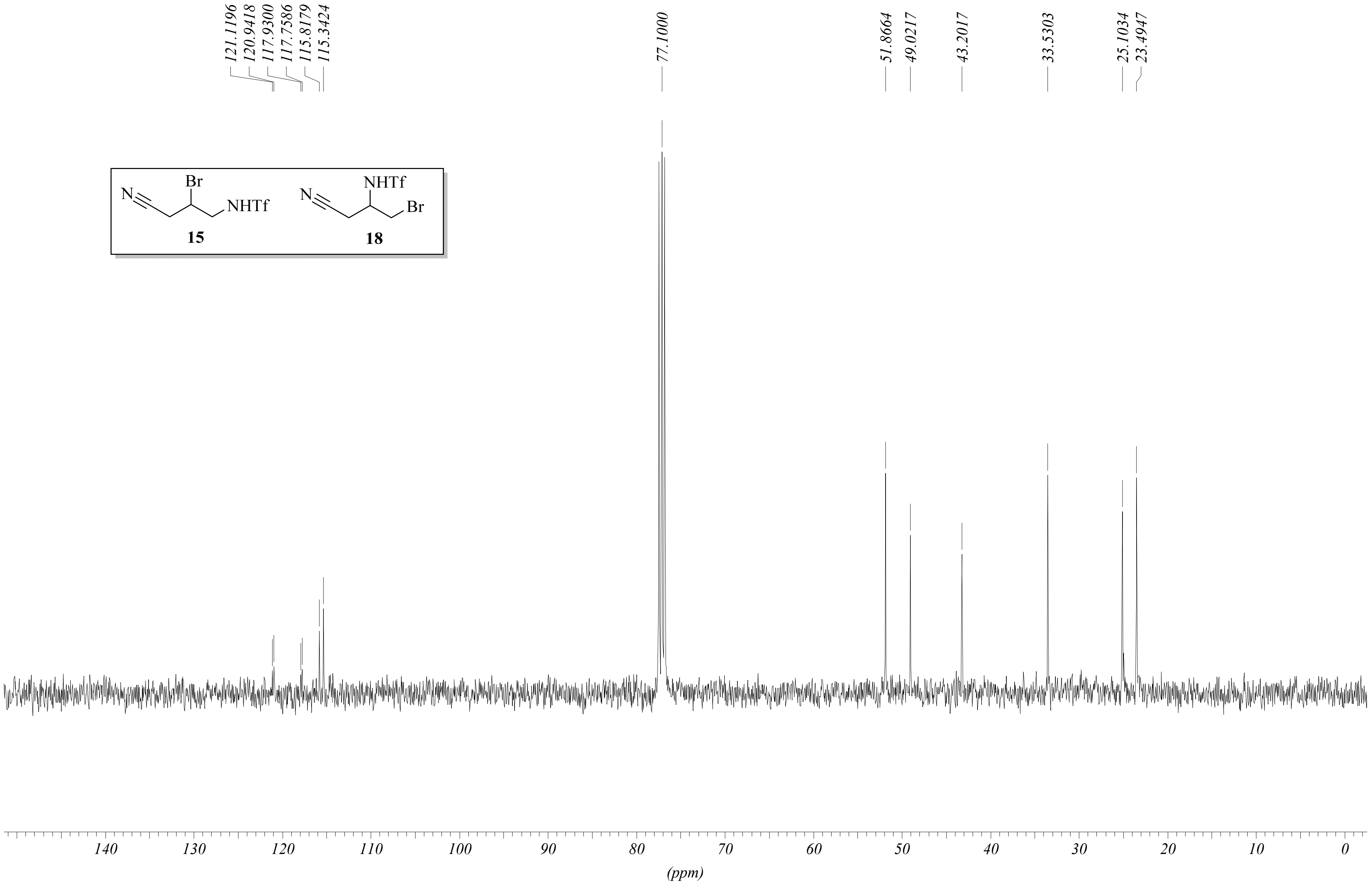

Figure S17. ^{1}H NMR spectrum of compound **17**

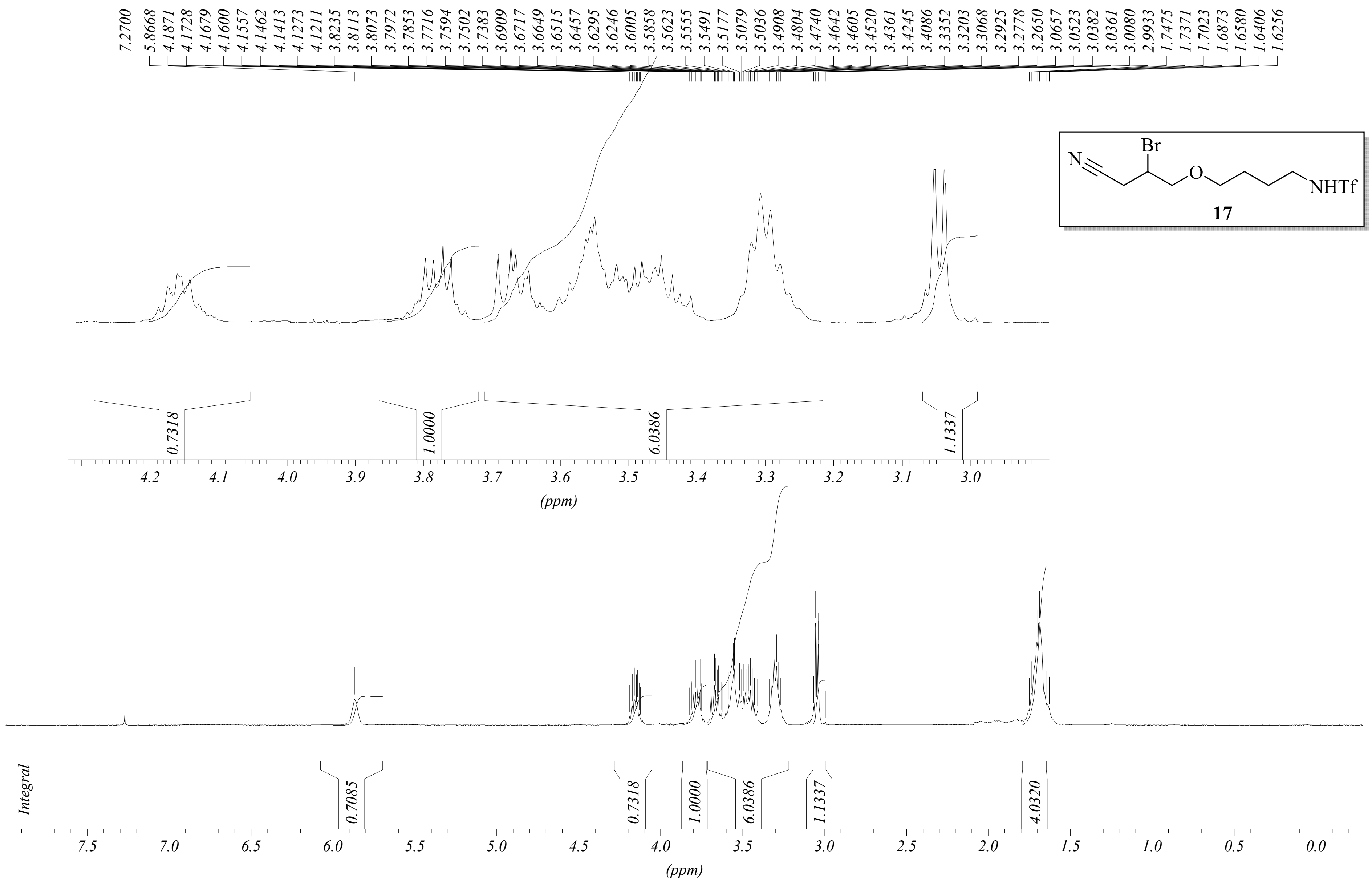

Figure S18. ^{13}C NMR spectrum of compound **17**

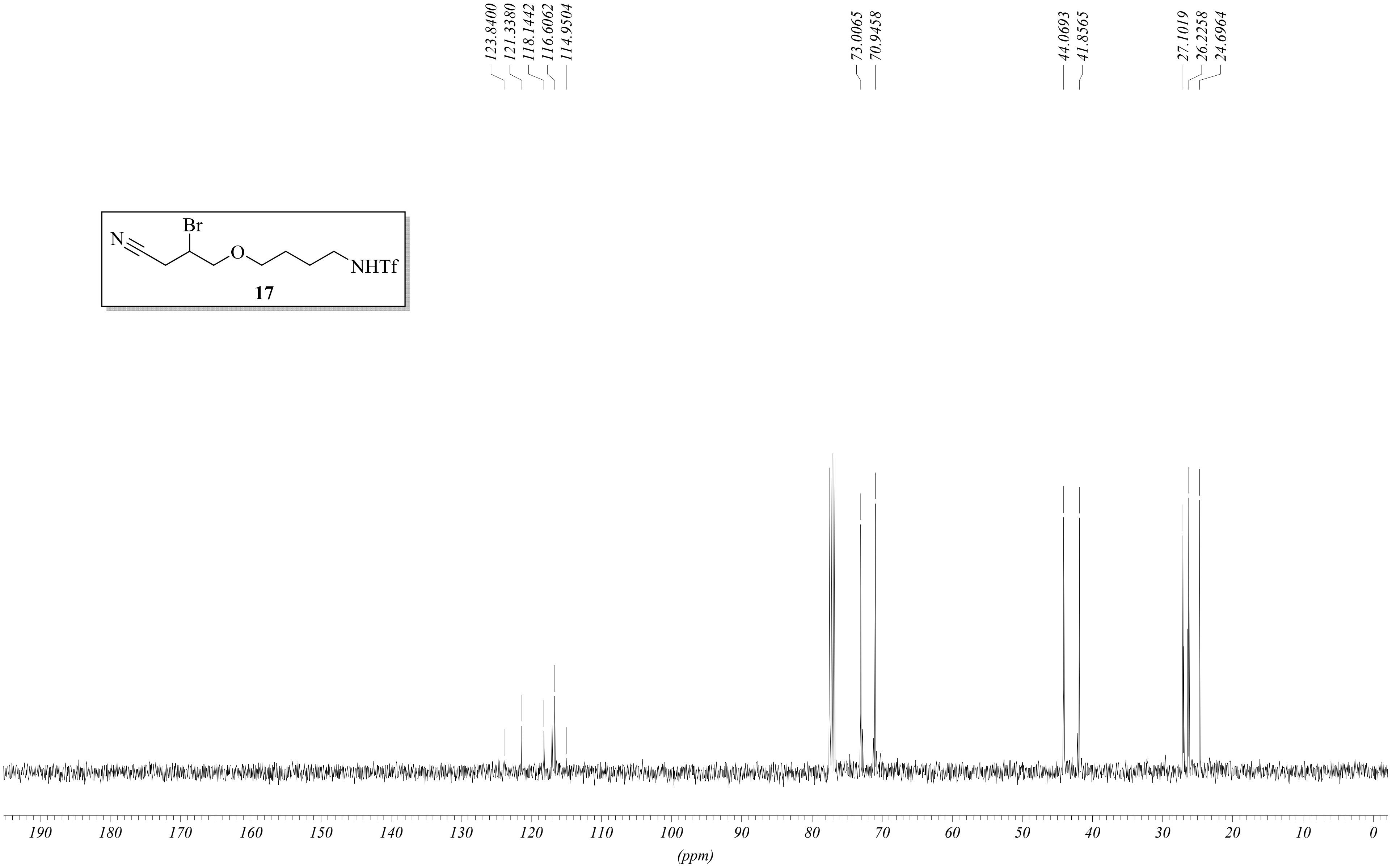

Figure S19. ^{1}H NMR spectrum of compound **19**

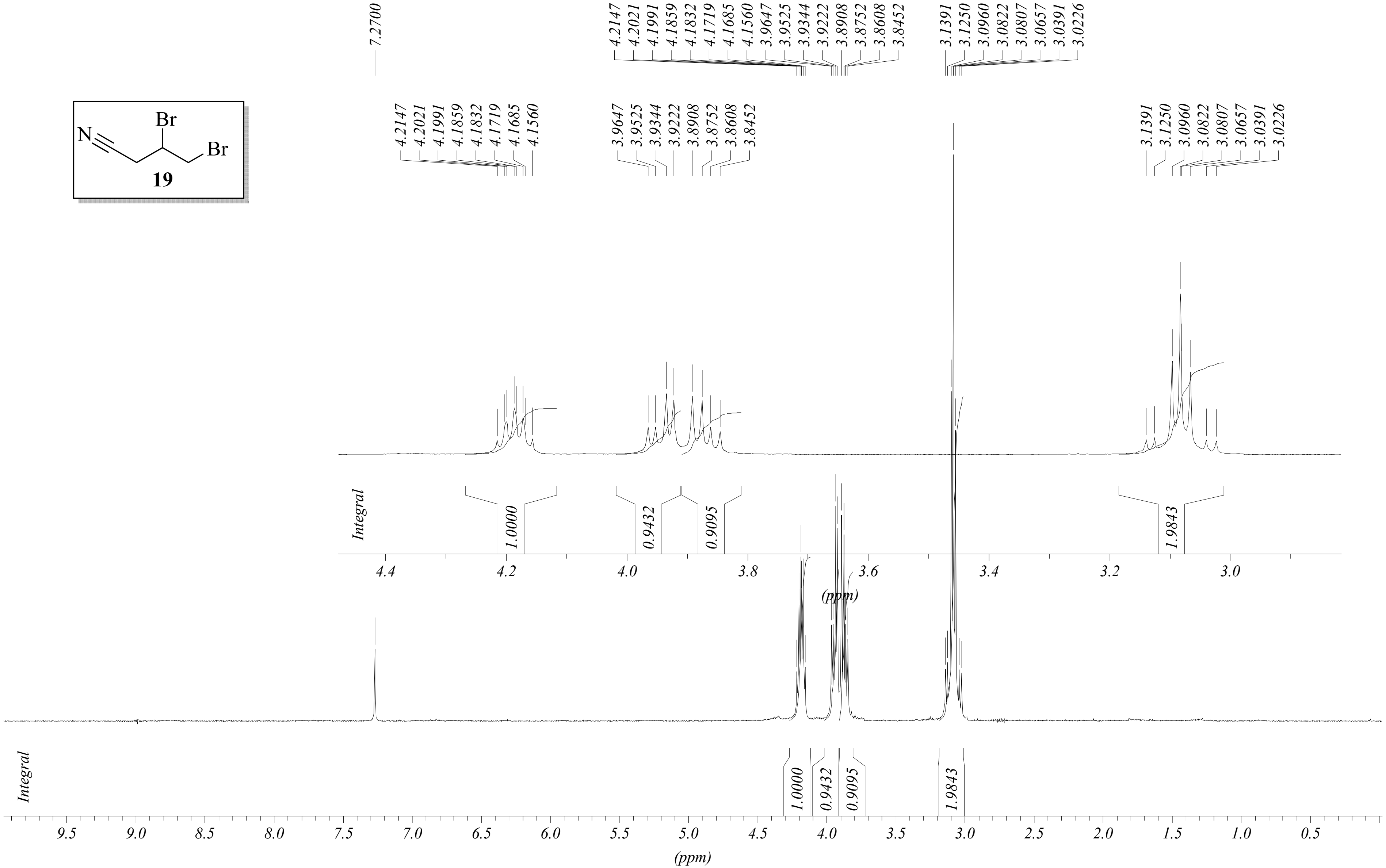

Figure S20. ^{13}C NMR spectrum of compound **19**

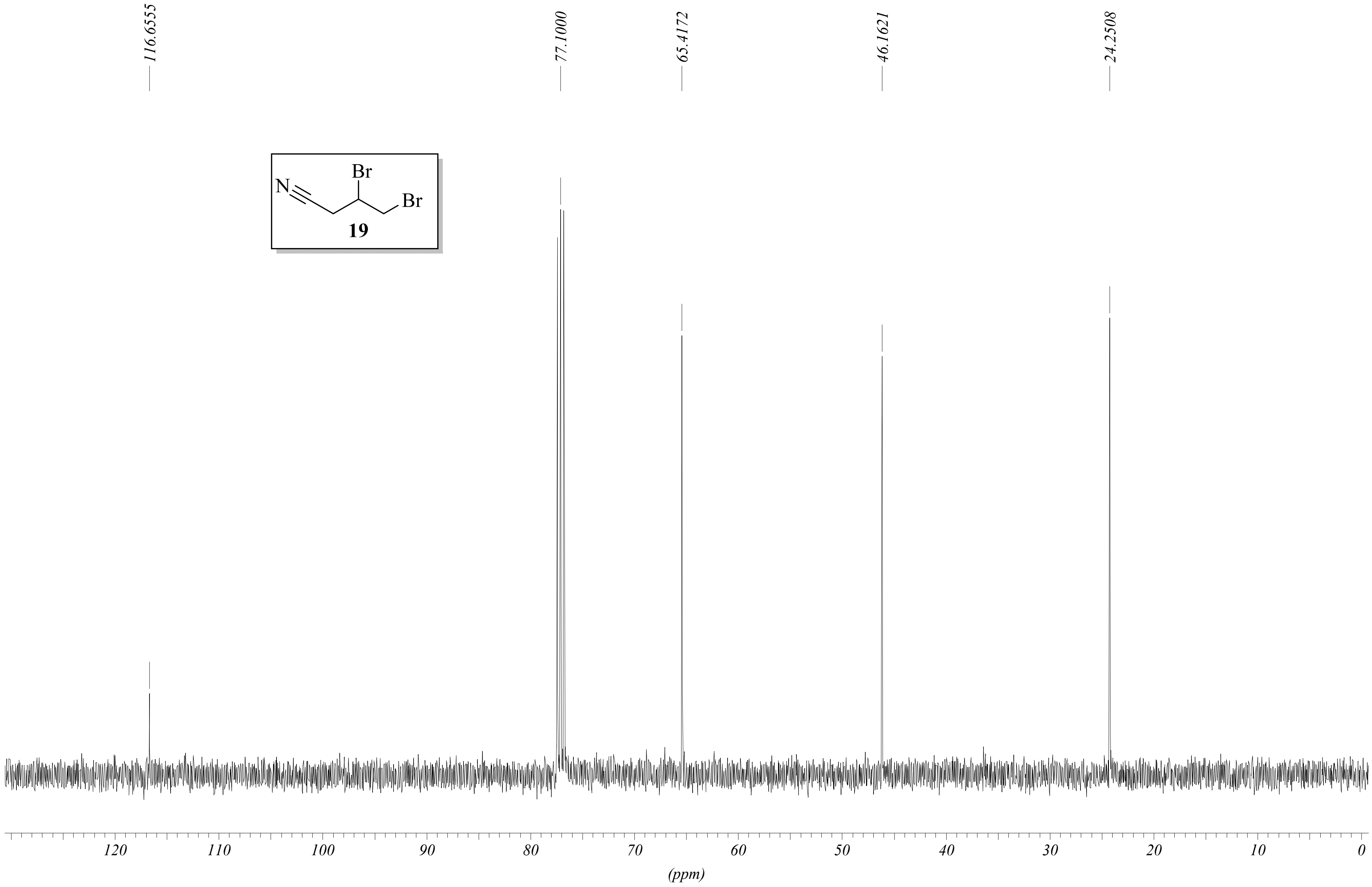

Figure S21. ^{1}H NMR spectrum of compound **20**

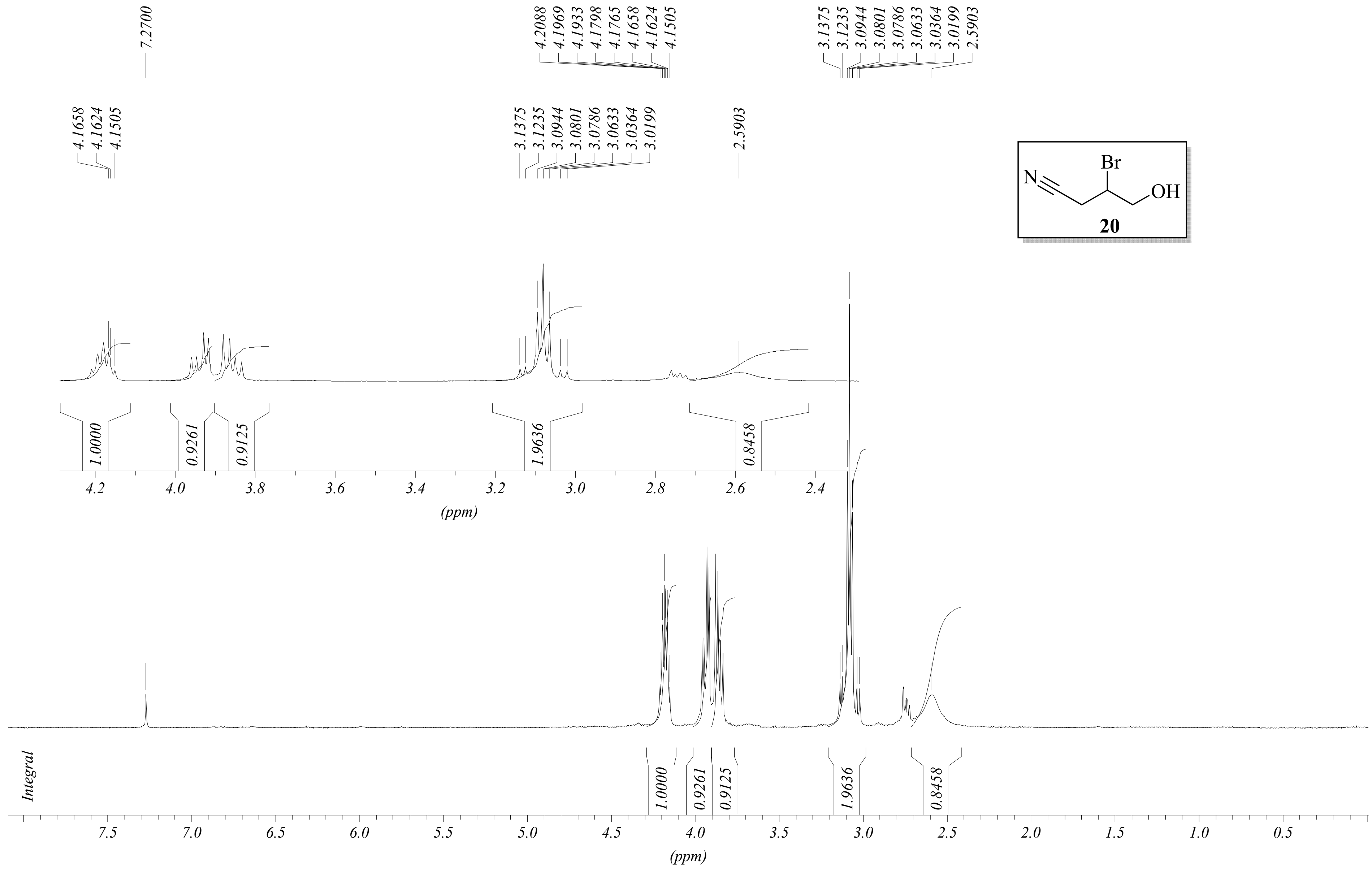

Figure S22. ^{13}C NMR spectrum of compound **20**

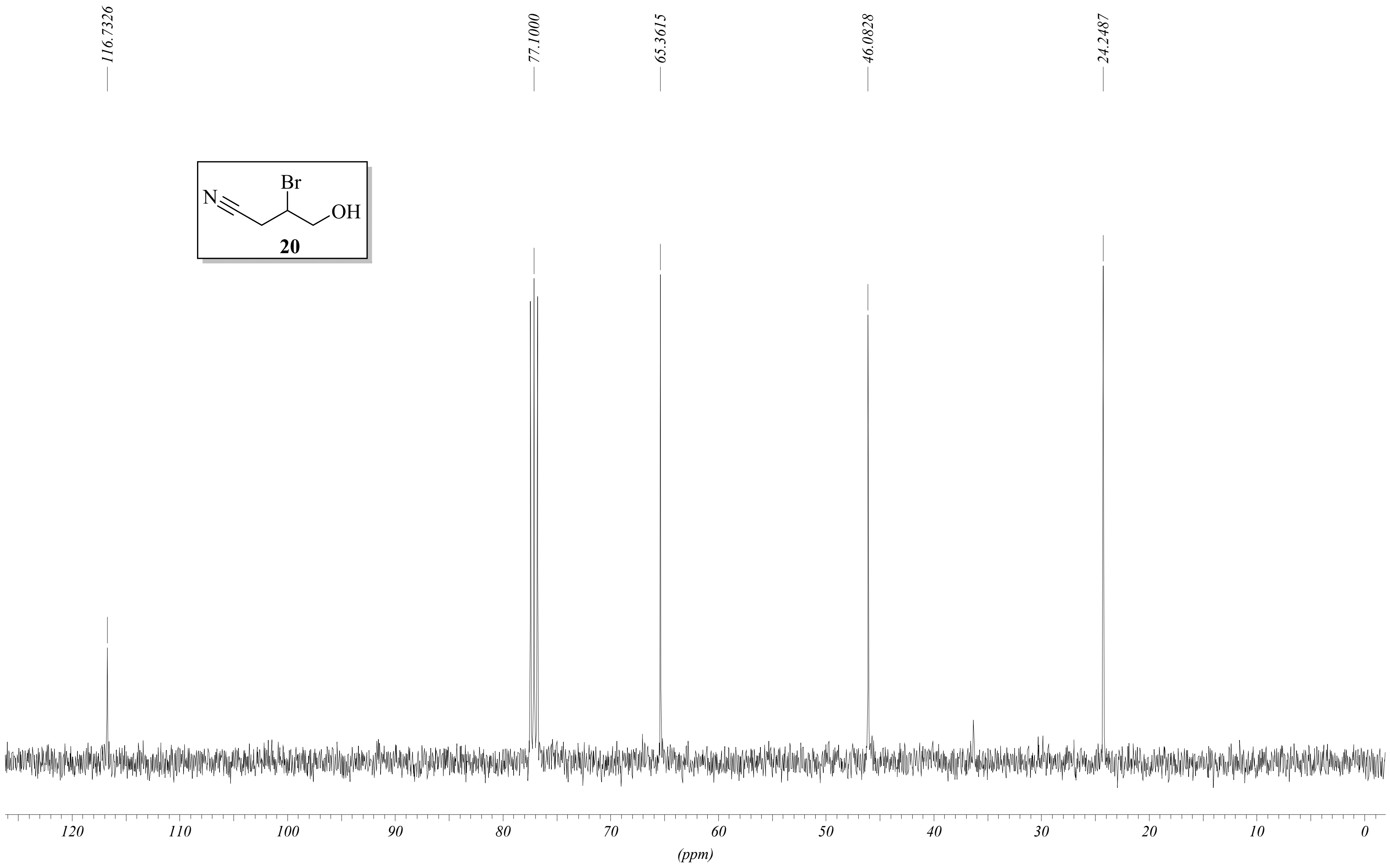

Figure S23. ^{1}H NMR spectrum of compound **21**

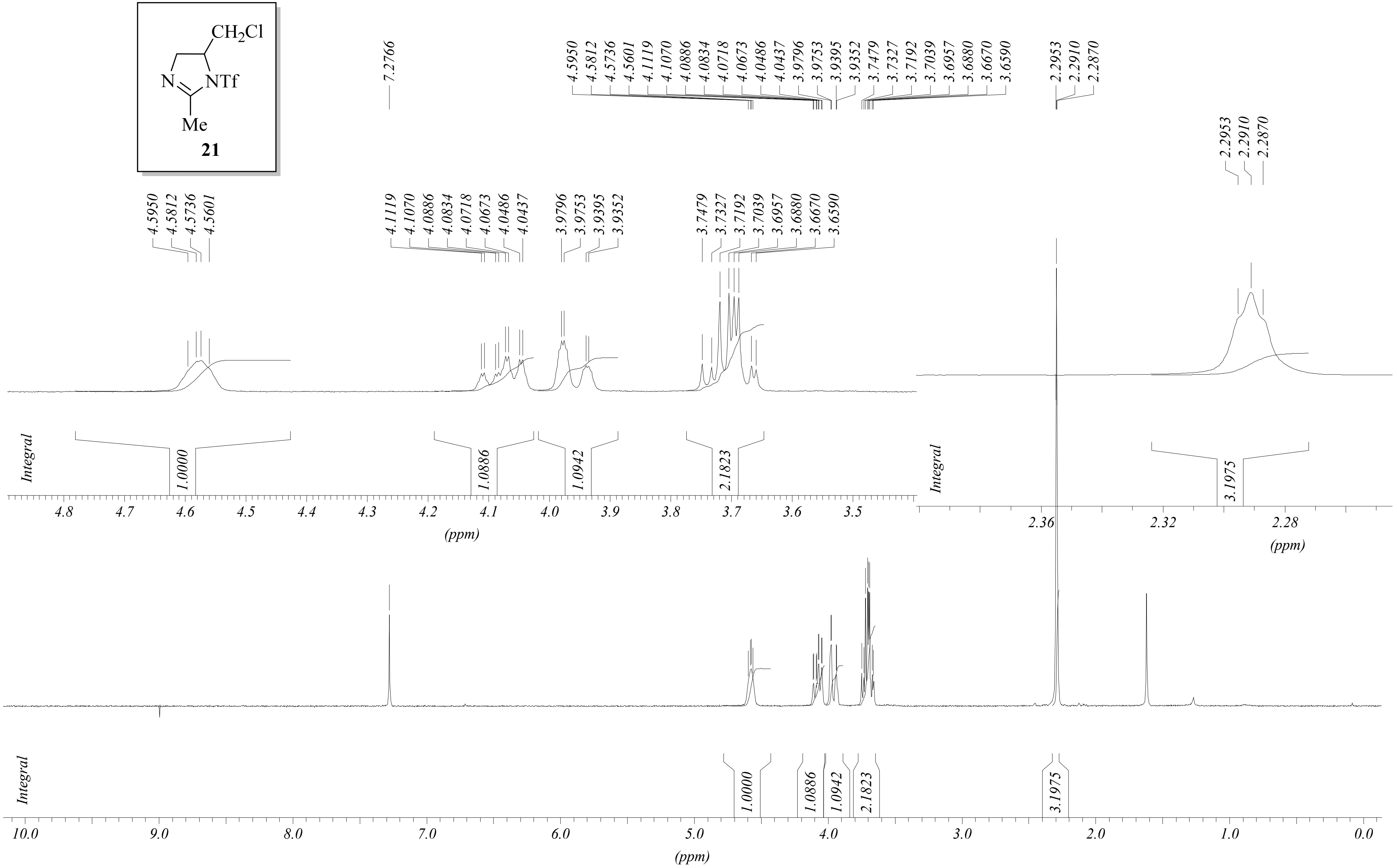

Figure S24. ^{13}C NMR spectrum of compound **21**

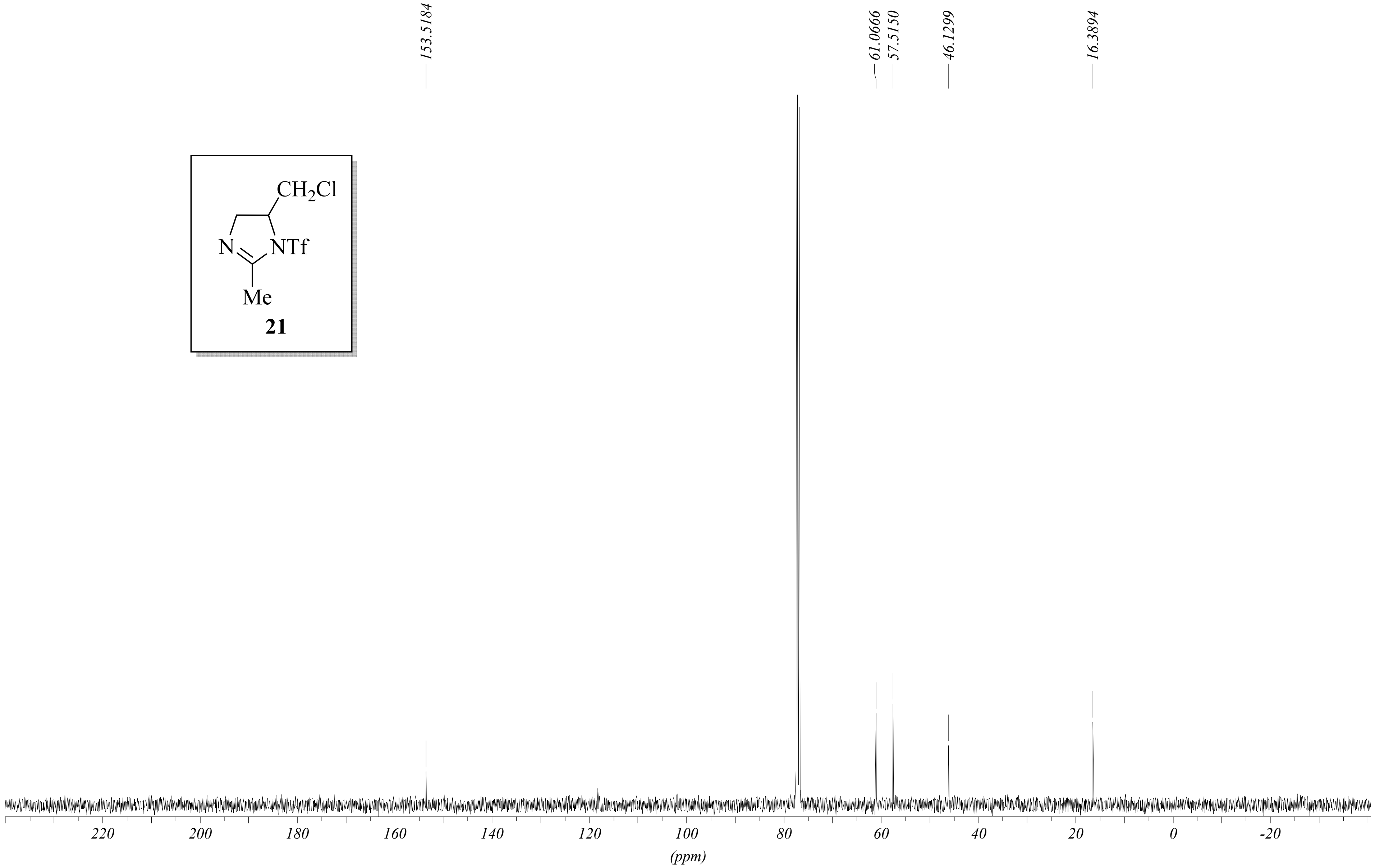

Figure S25. ^{1}H NMR spectrum of compound **22**

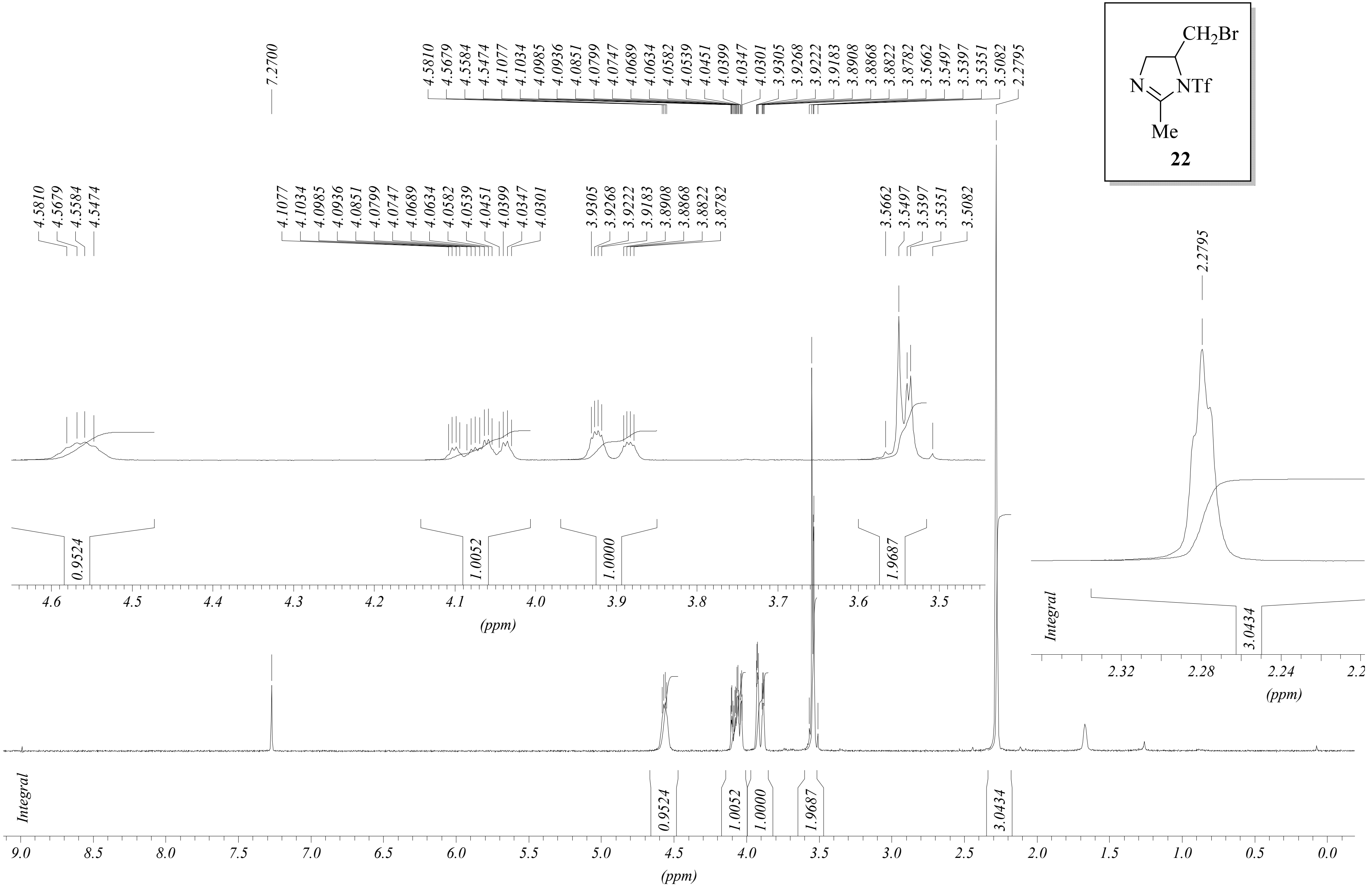

Figure S26. ^{13}C NMR spectrum of compound **22**

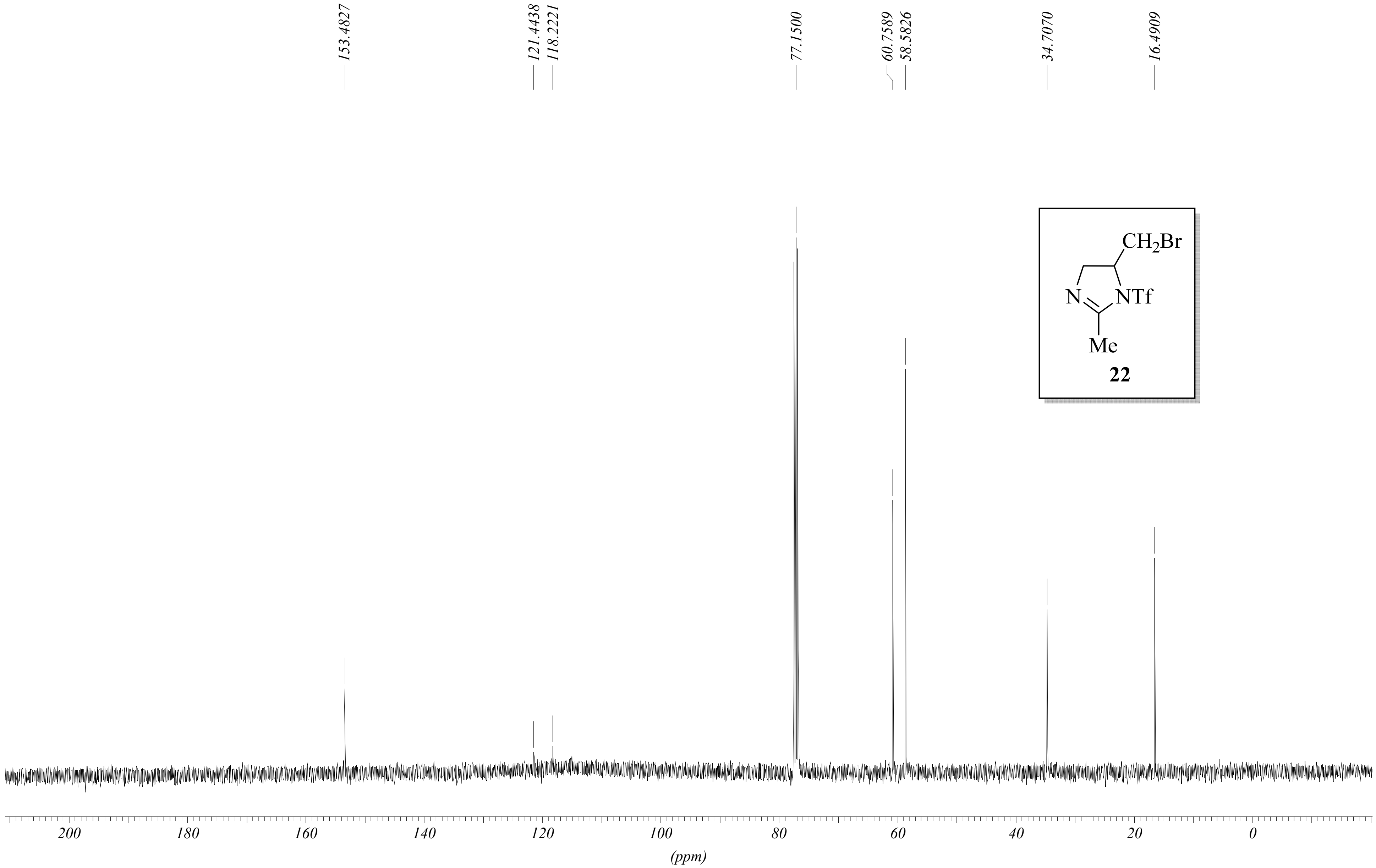

Figure S27. ^{1}H NMR spectrum of compound **23**

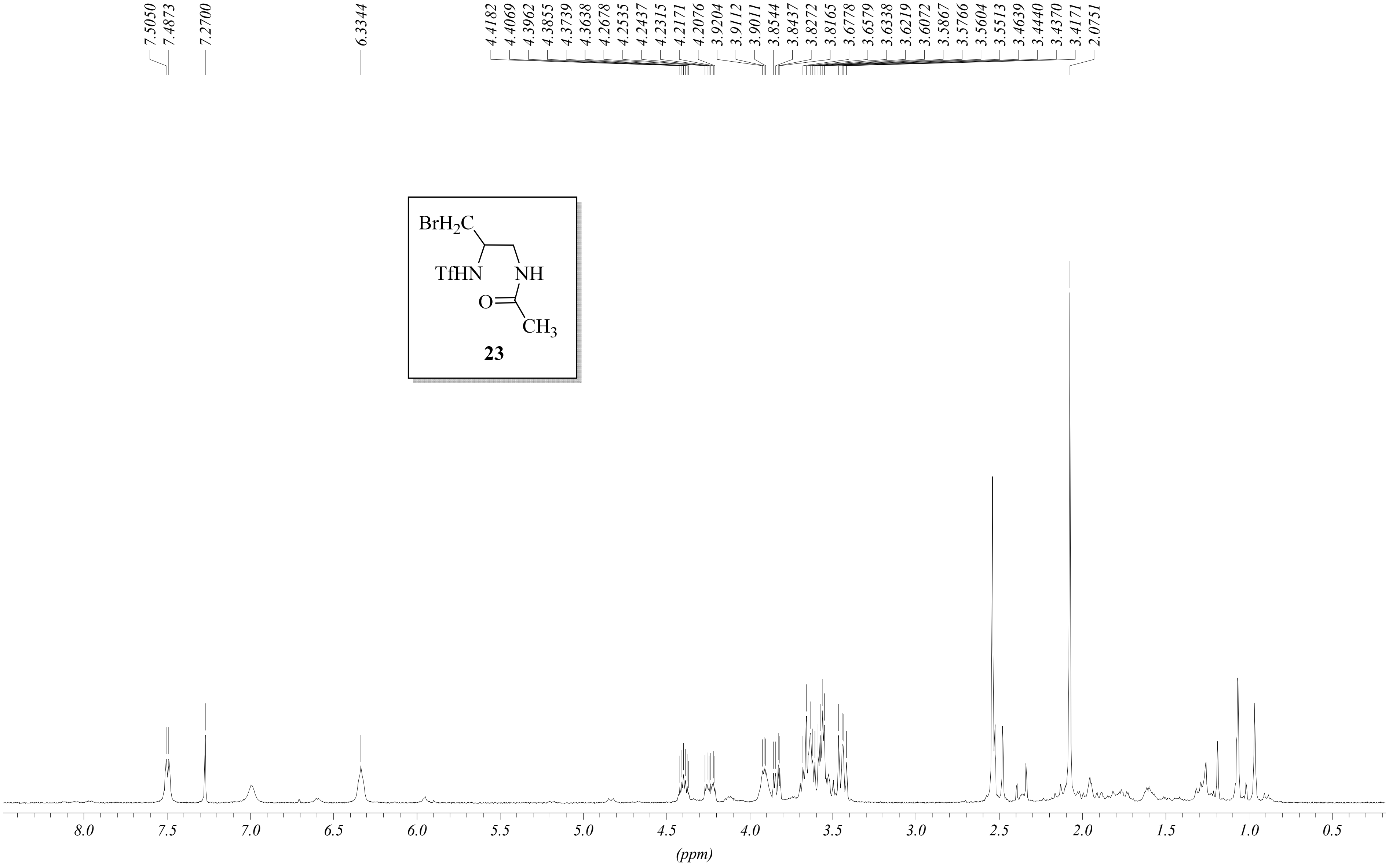

Figure S28. 13C NMR spectrum of compound **23**

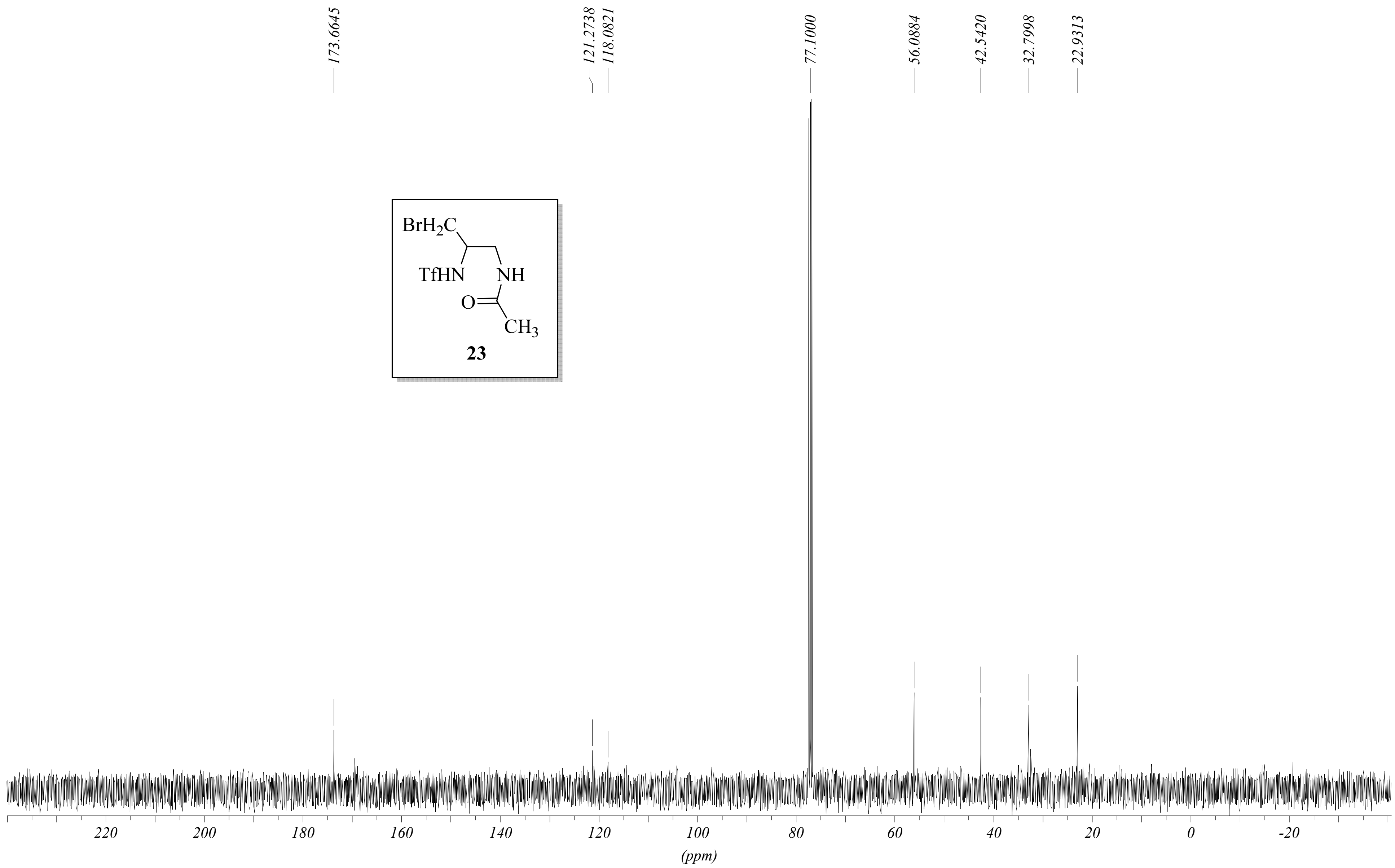

Figure S29. ^{1}H NMR spectrum of compound **25**

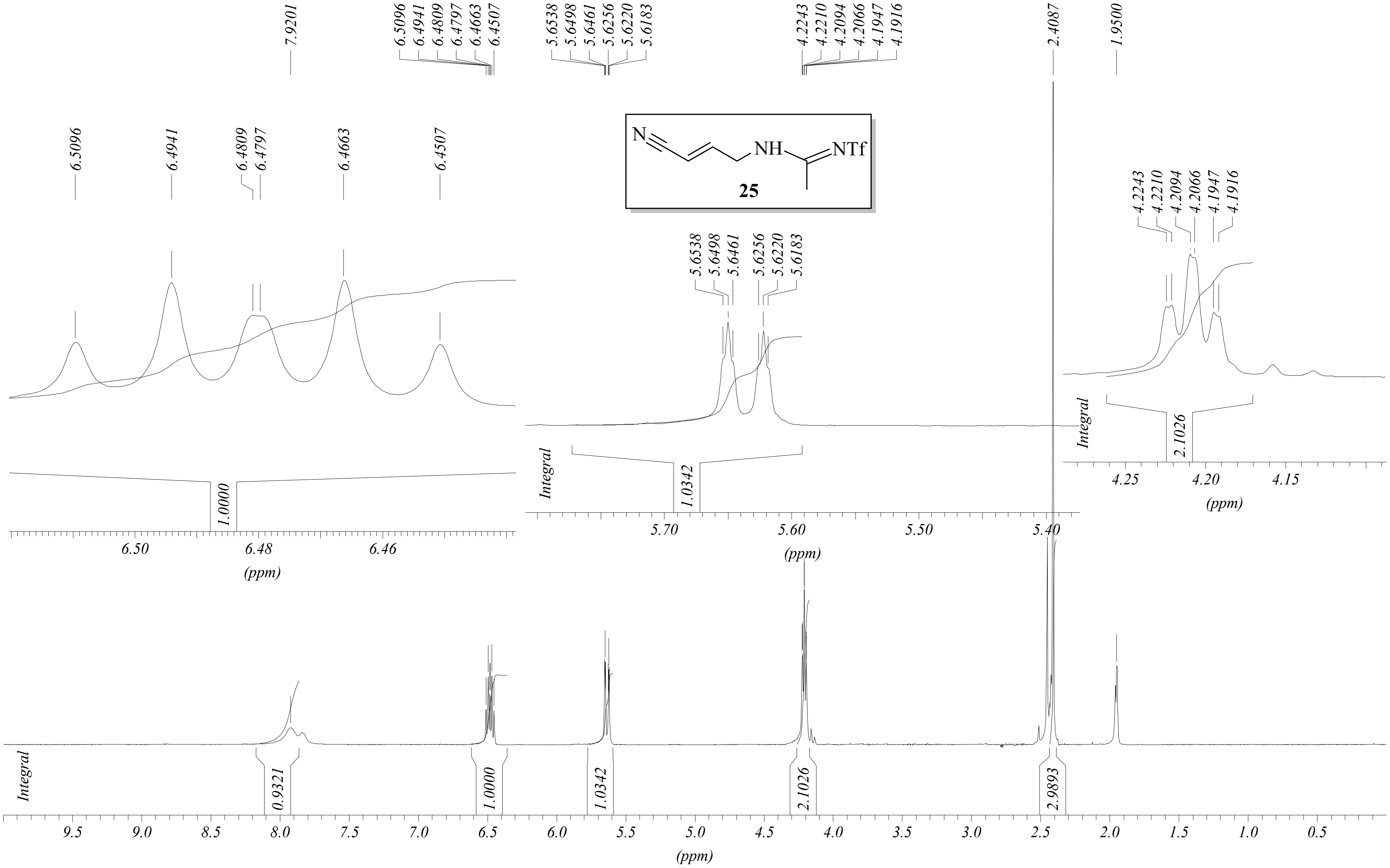

Figure S30. 2D NMR {^{1}H-^{1}H}-COSY spectrum of compound **25**

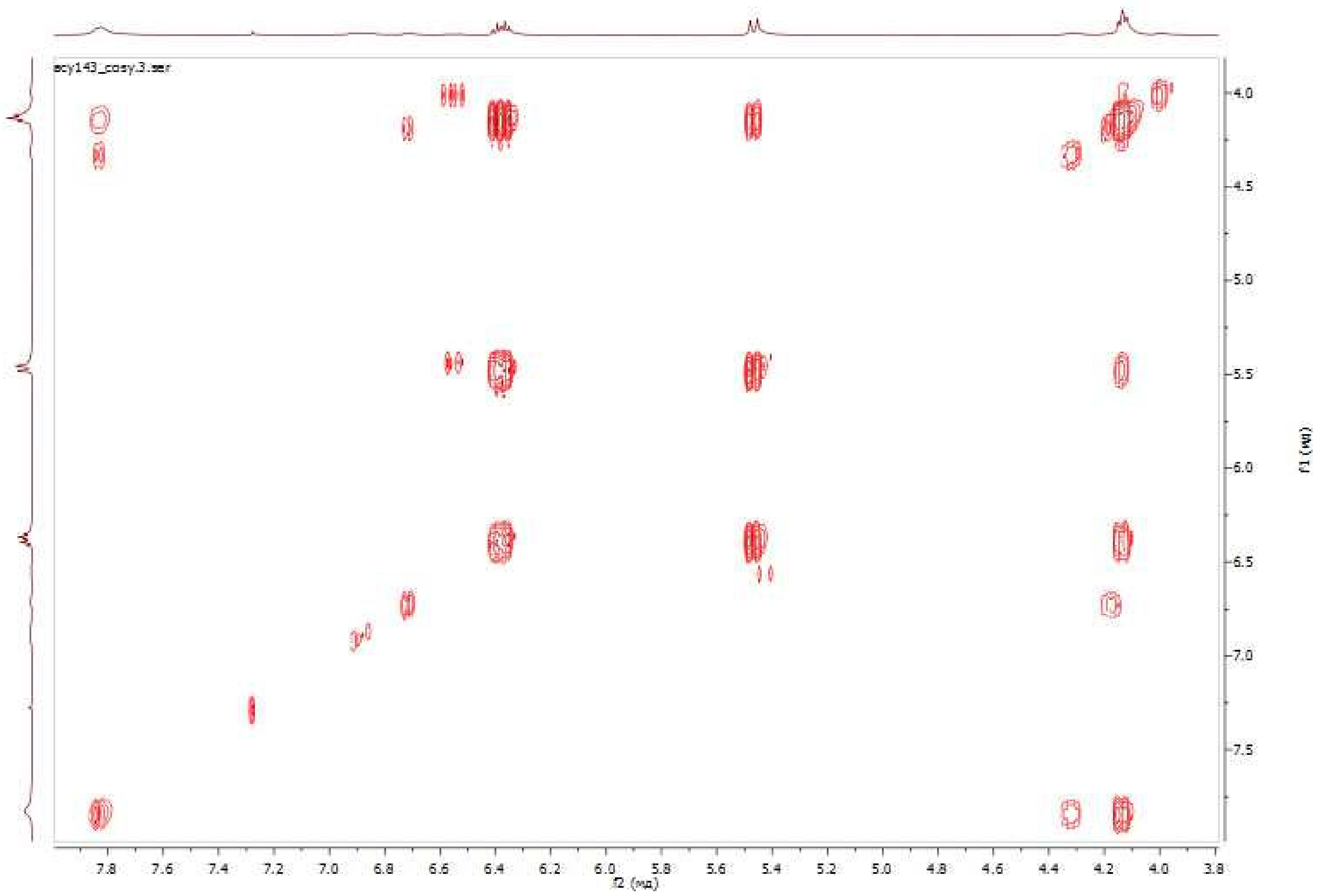

Figure S31. ^{13}C NMR spectrum of compound **25**

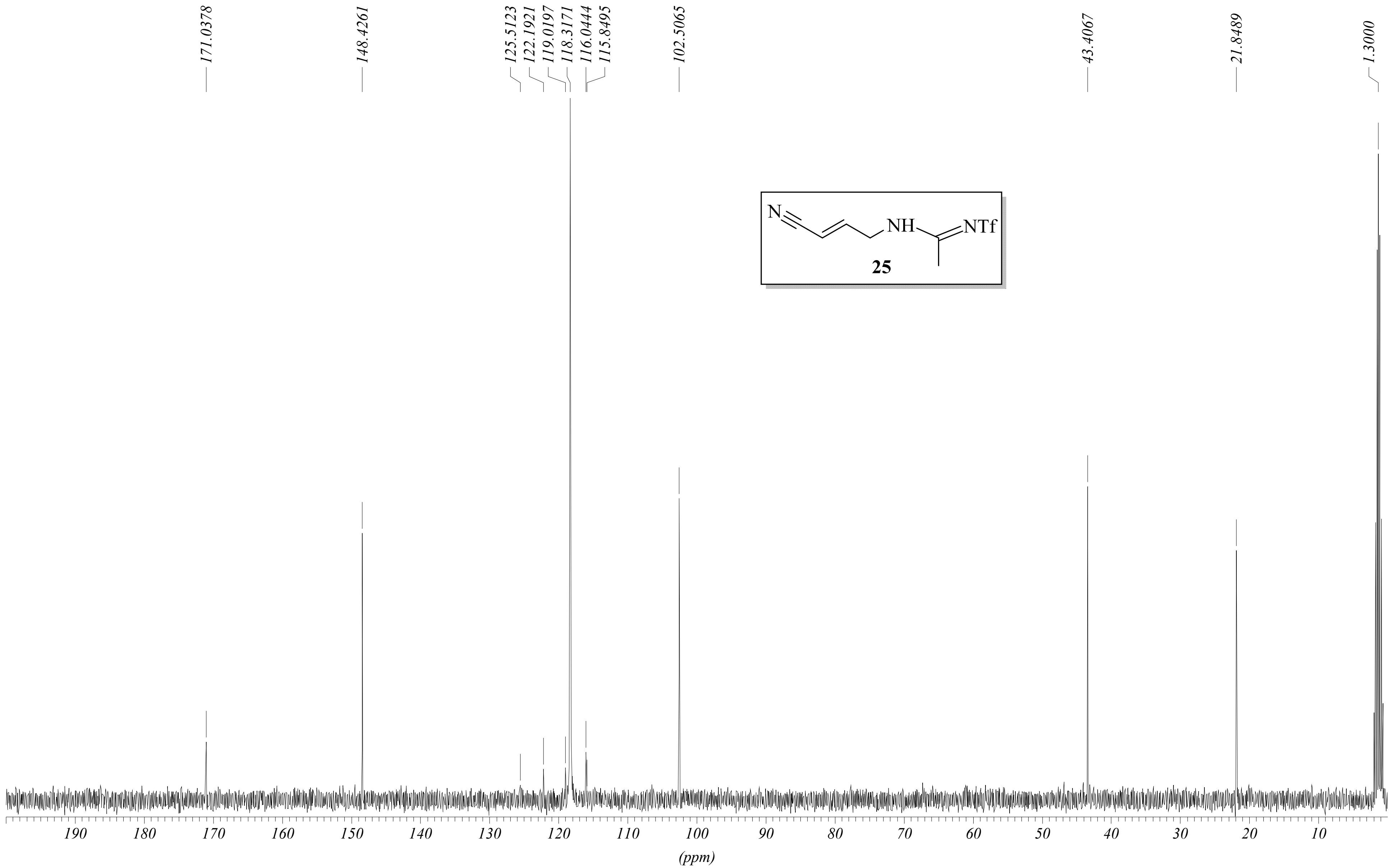

Figure S32. ^{1}H NMR spectrum of compound **26**

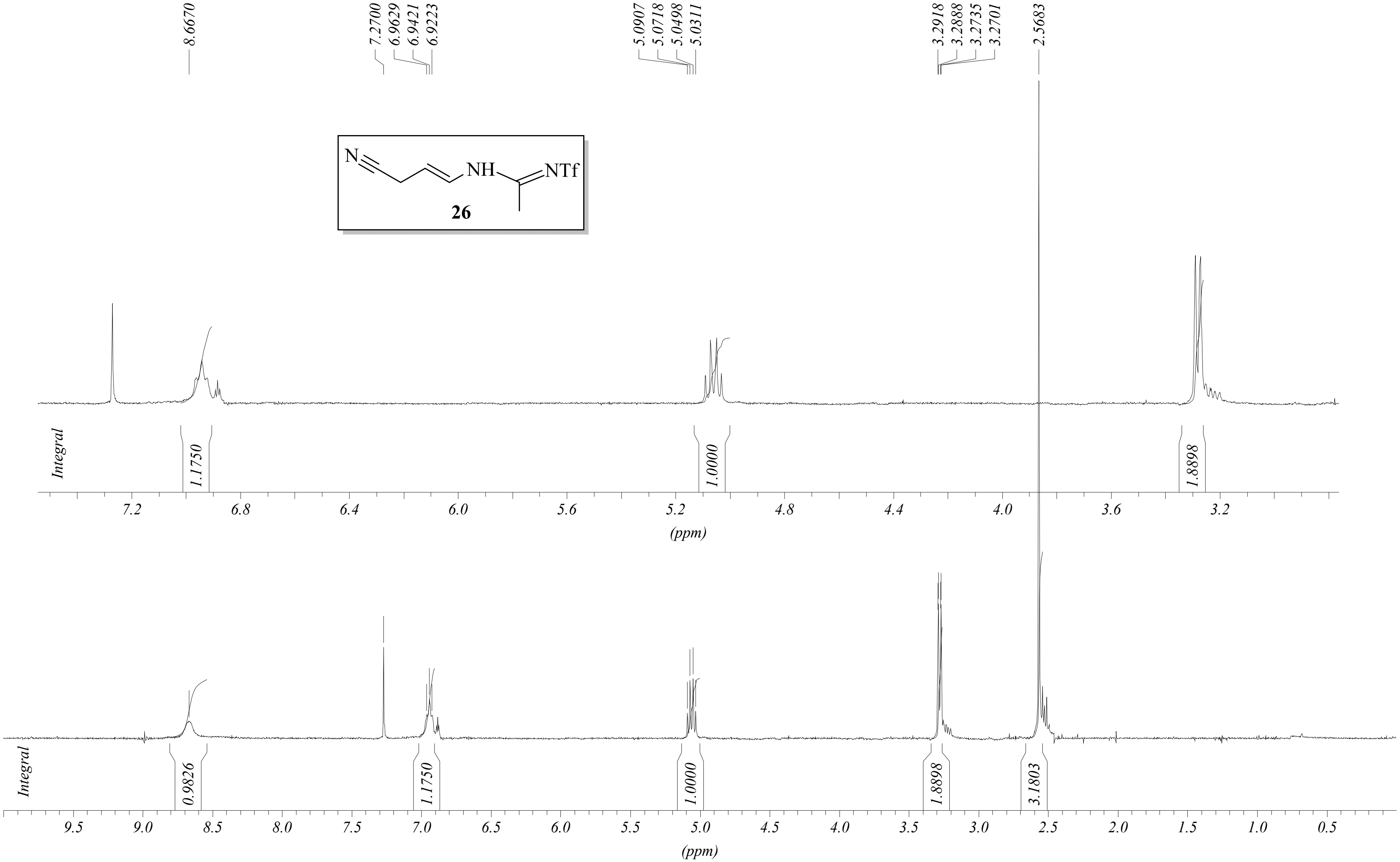

Figure S33. ^{13}C NMR spectrum of compound **26**

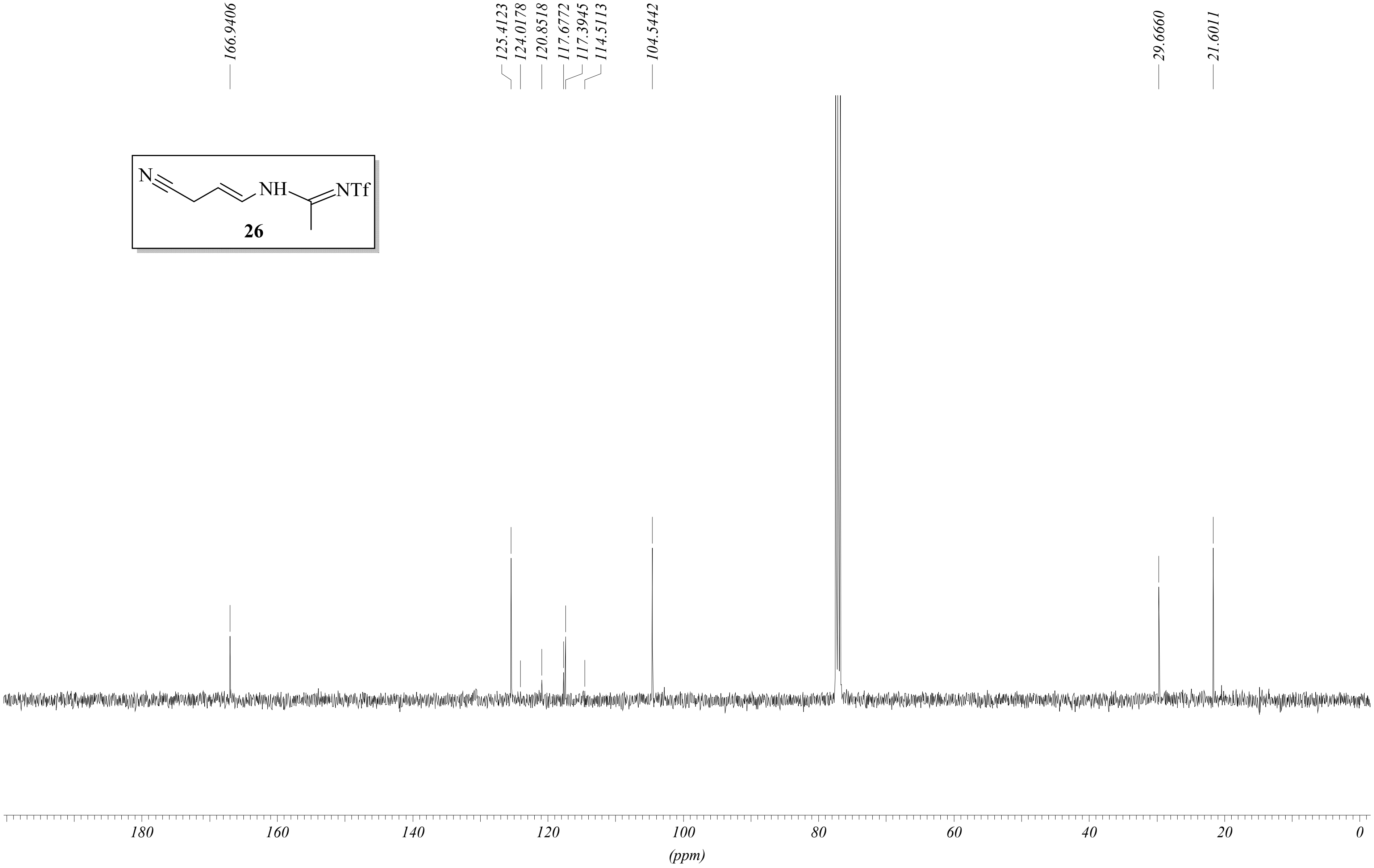

Figure S34. ¹H NMR spectrum of compounds **27** and **28**

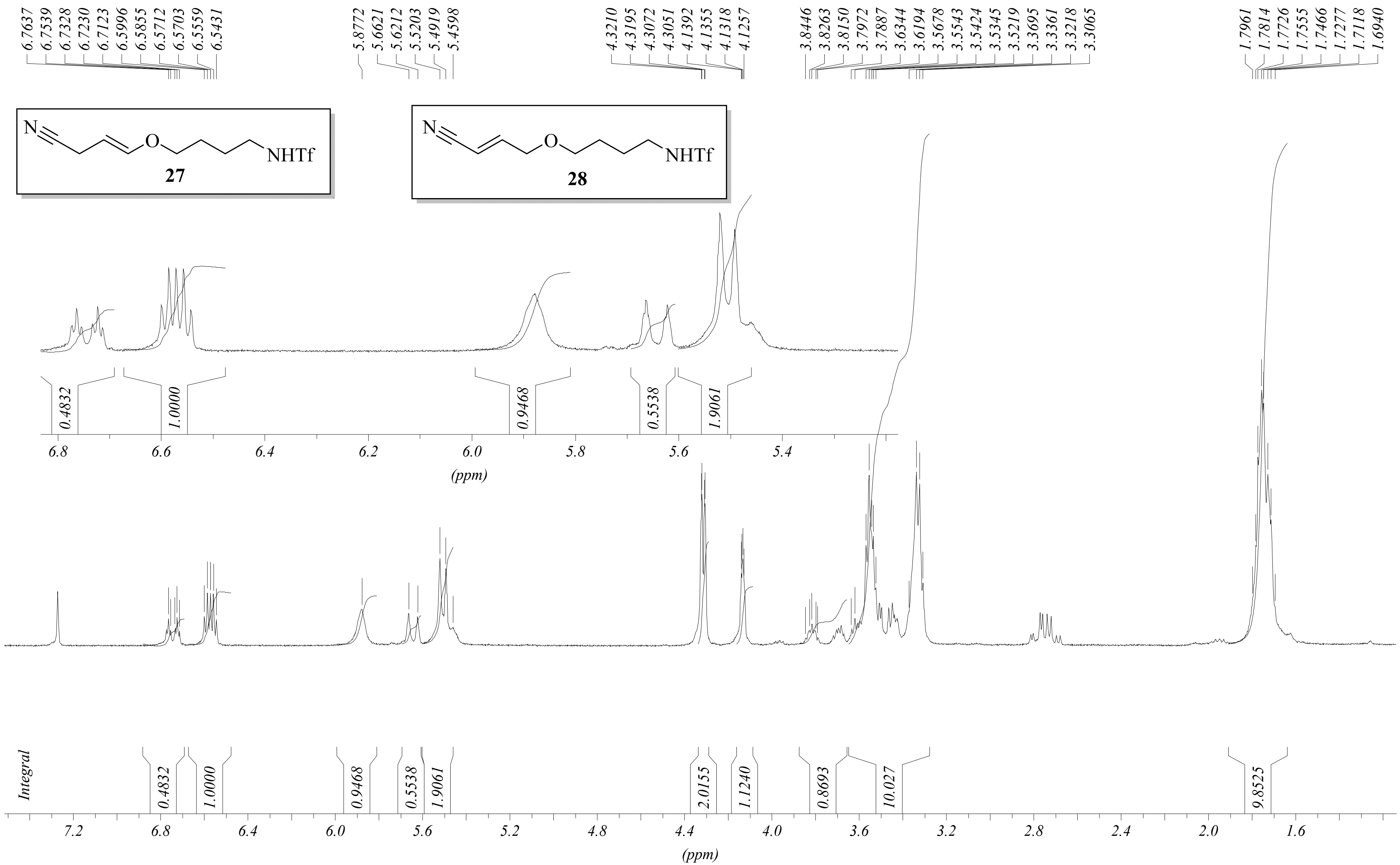

Figure S35. ^{13}C NMR spectrum of compound **27** and **28**

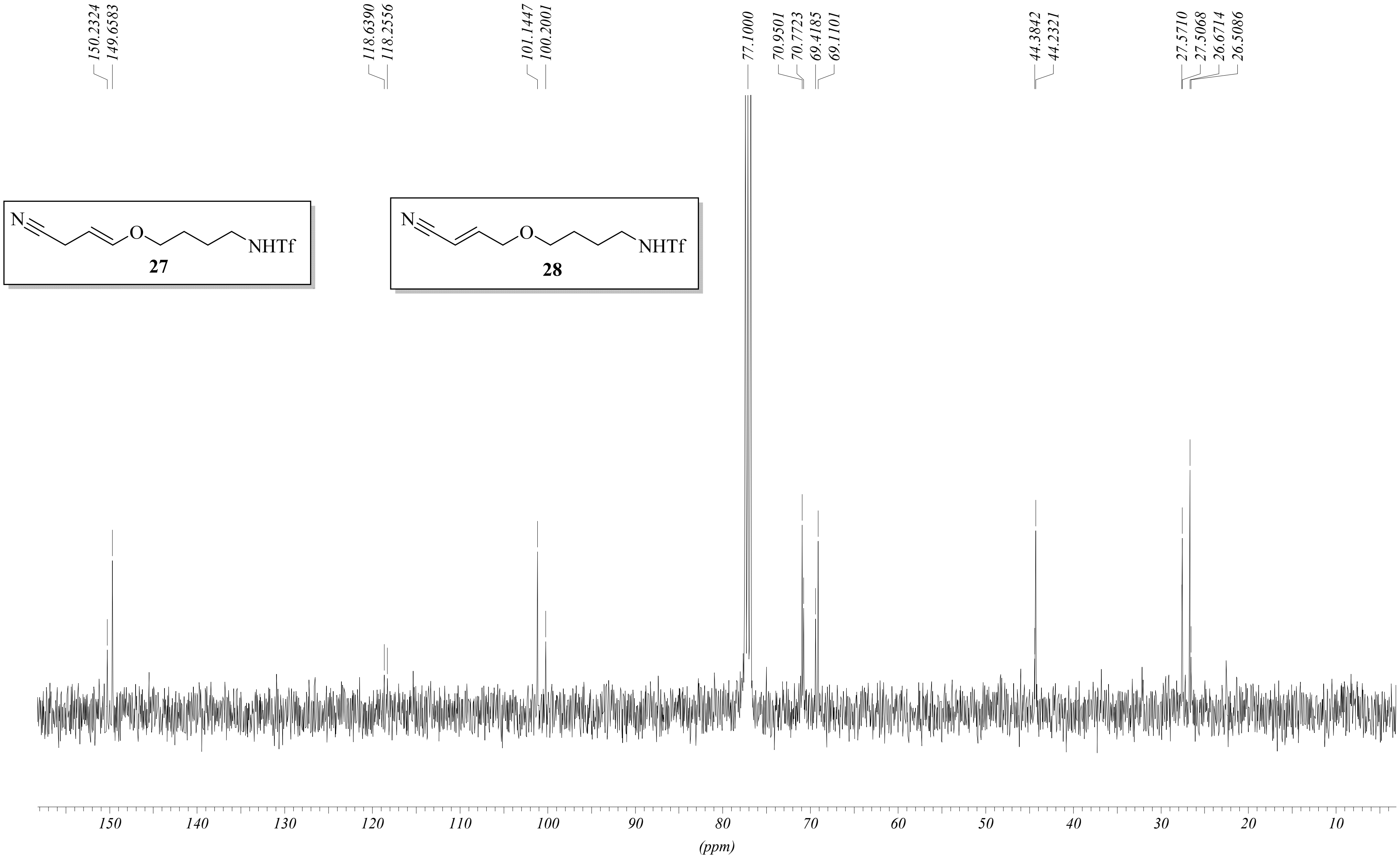

Figure S36. HRMS compound **4**

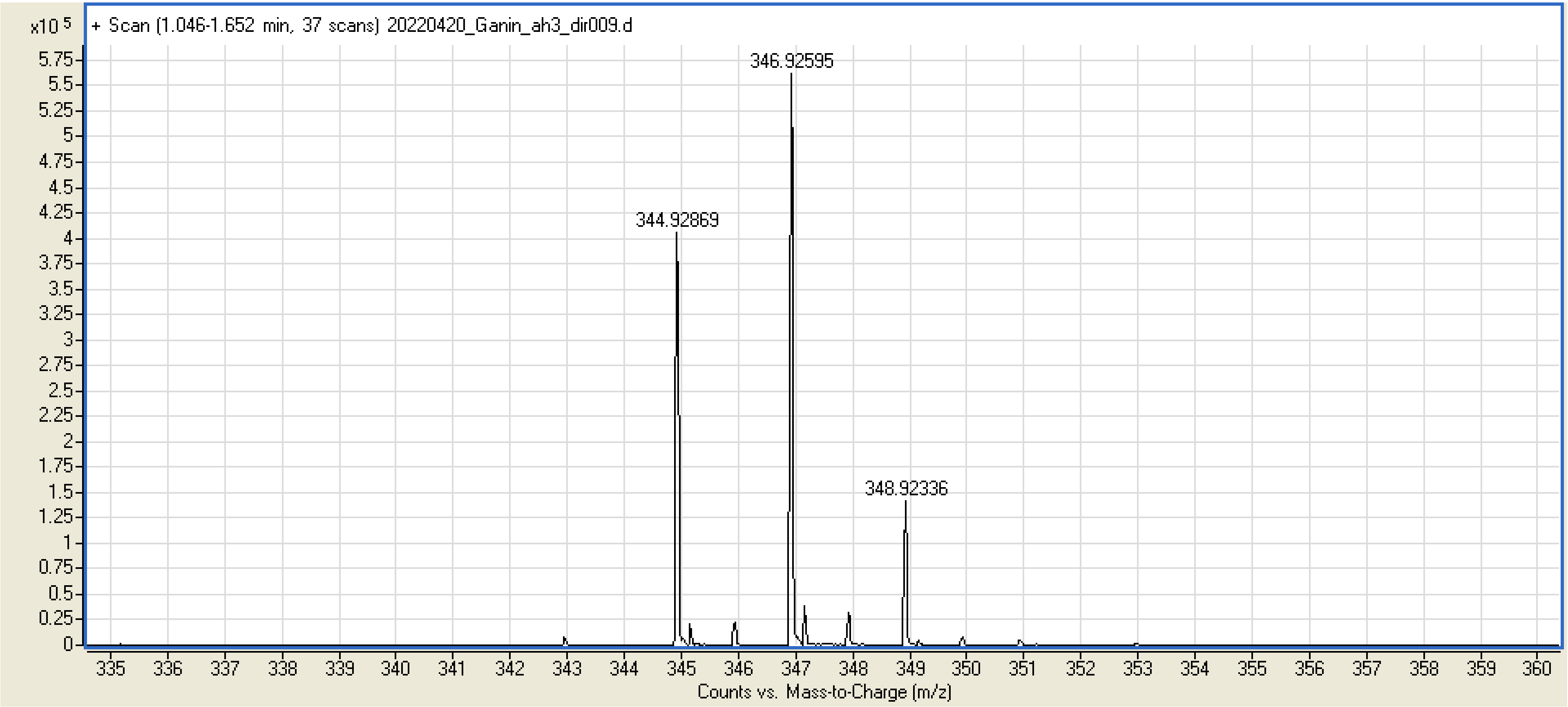

Figure S37. HRMS compound **14**

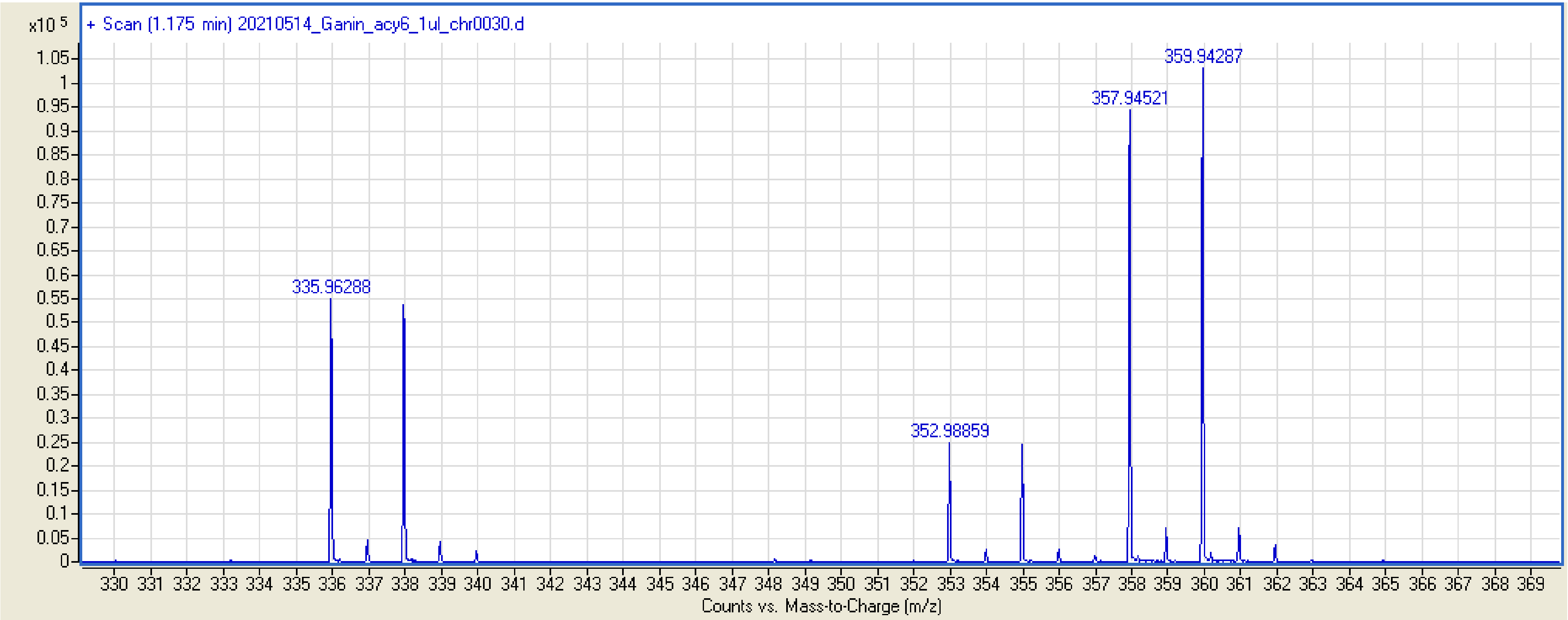

Figure S38. HRMS compound **25**

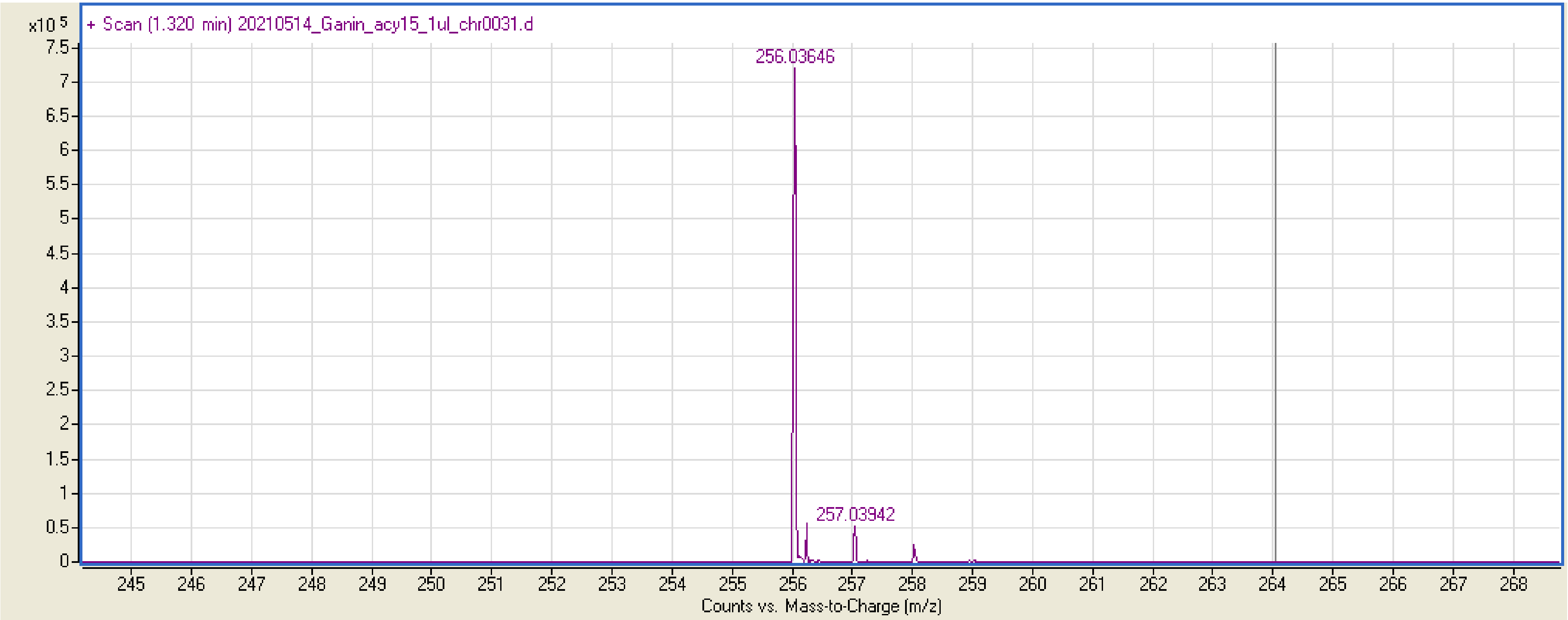

Supplement: Supplementary file 1 [file molecules-27-06910-s001.zip › molecules-1944736-supplementary.pdf]
